# The embryonic node behaves as an instructive stem cell niche for axial elongation

**DOI:** 10.1073/pnas.2108935119

**Published:** 2022-01-31

**Authors:** Tatiana Solovieva, Hui-Chun Lu, Adam Moverley, Nicolas Plachta, Claudio D. Stern

**Affiliations:** ^a^Department of Cell and Developmental Biology, University College London, WC1E 6BT London, United Kingdom;; ^b^Institute of Molecular Cell Biology, A*STAR, 138673 Proteos, Singapore

**Keywords:** Hensen’s node, stem cell niche, self-renewal, primitive streak, tail bud

## Abstract

In warm-blooded vertebrate embryos (mammals and birds), the axial tissues of the body form from a growth zone at the tail end, Hensen’s node, which generates neural, mesodermal, and endodermal structures along the midline. While most cells only pass through this region, the node has been suggested to contain a small population of resident stem cells. However, it is unknown whether the rest of the node constitutes an instructive niche that specifies this self-renewal behavior. Here, we use heterotopic transplantation of groups and single cells and show that cells not destined to enter the node can become resident and self-renew. Long-term resident cells are restricted to the posterior part of the node and single-cell RNA-sequencing reveals that the majority of these resident cells preferentially express G2/M phase cell-cycle–related genes. These results provide strong evidence that the node functions as a niche to maintain self-renewal of axial progenitors.

## Significance

Previous studies have suggested that the amniote node (Hensen’s node) contains a small population of self-renewing resident cells whose progeny progressively lay down axial tissues, including notochord and somites. This can only be demonstrated definitively at the level of single cells. Here we ask whether the node is an environment that can confer this behavior on cells that enter it. We challenge single cells in vivo and mRNA-profile these cells to demonstrate that the node can indeed do this, and thus show that the node acts as an instructive niche.

**Fig. 1.**
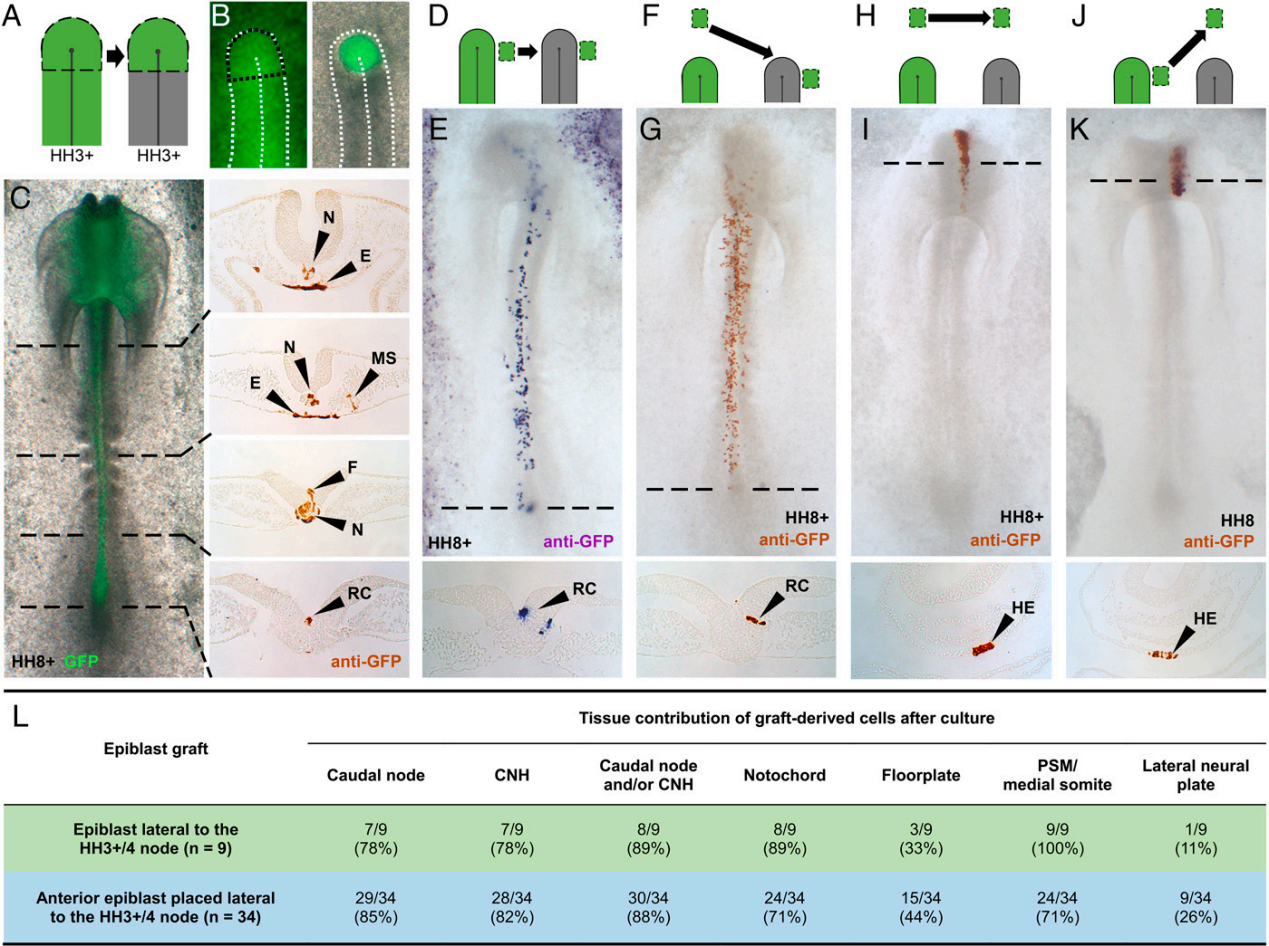
The node confers resident behavior. (*A*–*C*) Node replacement using a GFP donor showing normal node axial fates. (*D* and *E*) Epiblast lateral to the HH3+/4 node ingresses into it and gives rise to the axis and to regressing node as resident cells. (*F* and *G*) Anterior epiblast not normally fated to enter the node behaves as lateral epiblast when forced to do so. (*H* and *I*) Anterior epiblast normally gives rise to head structures. (*J* and *K*) Lateral epiblast no longer gives rise to node-derived axial structures when prevented from entering the node. (*L*) Quantifying tissue contribution of lateral (*D*, green) versus anterior *(F*, blue) epiblast grafts to the host. E, endoderm; F, floorplate; MS, medial-somite; N, notochord; RC, resident cell. Transverse dashed lines show levels of accompanying sections. The field of view of the wholemount images (*C*, *E*, *G*, *I*, *K*) is approximately 2 mm x 5 mm.

In higher vertebrate embryos the body axis forms in head-to-tail direction from a growth zone at the tail end, which is present from gastrula stages through to the end of axis elongation, several days later. Hensen’s node is part of this growth zone. Rather than defining a distinct cell population arising very early in development, the node represents a dynamic region at the tip of the primitive streak, which appears as a morphological “node” from HH4 (1) in chick. The initial cells that make up this region are derived from two distinct cell populations, which meet at the tip of the elongating primitive streak (HH3 to 3+ in chick) (2–4). These are then joined by cells from the epiblast lateral to the anterior streak and node (5) (at stages HH3+ to HH4) and from the primitive streak immediately caudal to the node during regression (from stage HH5) (6, 7). Although ingression of cells from adjacent epiblast along most of the length of the streak continues later into development (6), this ceases at the level of the node by HH4+ (5, 8, 9). After stage 5, the node begins to regress caudally (7), while cells exit the node to lay down the midline of the developing head–tail axis, contributing to axial (notochord) and paraxial (medial somite) mesoderm, definitive endoderm, and neural midline (floorplate) tissues (Fig. 1 *A*–*C*) (5, 10–12).

Therefore, most cells pass transiently though the node, temporarily gaining a node-like gene-expression signature, which they lose upon leaving the node (5). However, transplantation of cell groups and fate-mapping experiments in chick (10, 13–15) and mouse (16–20) during early development have suggested that the node may also contain a few resident self-renewing cells that persist within the node during axial elongation, while other cells leave (Fig. 1*C*, “RC”). In particular, labeling of single cells in the node has provided a few examples of cells that contribute to midline structures and appear to self-renew because one or more cells remain at the site of labeling after some progeny have left (10, 17, 21, 22). At a cell-population level, grafts of groups of cells transplanted repeatedly between older and younger tailbud regions can contribute to midline structures over two or more hosts, while again some cells remain in the tailbud (14, 19). These findings have led to the idea that some cells in the node (most likely a very small subset) may have the ability to self-renew, perhaps indefinitely, thus displaying stem cell behavior.

Are the self-renewing cells a special population that arose in earlier development, or might the node act as an environment (niche) (23–25) that captures a subset of the cells that enter it and instructs them to become resident and acquire self-renewal behavior and act as stem cells (26–28)? To demonstrate self-renewal and to test whether the node is an instructive stem cell niche, it is critical to test whether an individual cell can acquire this behavior when introduced to the node environment; this has not yet been attempted. Here we address this question using transplantation of groups of cells and of single cells in vivo and single-cell RNA sequencing (scRNA-seq). We find that the tip of the primitive streak is able to impart notochord and somite identity to most or all cells that enter it, while capturing a small subset to become resident and acquire self-renewal behavior. Cells from epiblast that would never have entered the node region during normal development are able to read these cues. We also define the developmental stage at which epiblast cells lose competence to respond to node signals. Long-term resident cells are preferentially located in the posterior part of the node, and display enriched expression of G2/M cell cycle markers.

## Results

### Nonnode Cells Can Become Resident.

To test whether the node environment can impart resident behavior onto other cells, we grafted a very small piece of anterior epiblast (which never normally enters the node) (4, 11, 29, 30) to a position adjacent to the HH3+/4 node, so that transplanted cells would be carried into the node by gastrulation movements (Fig. 1*F* and *SI Appendix*, Fig. S1 *A*–*C*). Graft-derived cells (from a transgenic GFP donor) give rise to axial tissues and express appropriate molecular markers of node, notochord, and somite (Fig. 1*G* and *SI Appendix*, Fig. S1 *D*–*L*). Importantly, the contribution of this anterior epiblast to cells with resident behavior (88%, *n* = 30 of 34) is similar to that of the lateral epiblast (89%, *n* = 8 of 9), which does normally enter the node (Fig. 1 *D*, *E*, and *L*). These results show that the node can confer resident behavior and axial identity to epiblast cells that would not otherwise have done so. However, while both anterior and lateral grafts generated resident cells in the node that expressed node markers, such as Chordin and Foxa2 (*SI Appendix*, Fig. S1 *D*–*I*), the contribution of anterior epiblast grafts to axial mesodermal derivatives (notochord and medial somite) was less than that of lateral epiblast, and the contribution of the former to axial neural derivatives (floorplate and lateral neural plate) was greater (Fig. 1*L*). This bias of anterior epiblast toward neural and away from mesodermal axial derivatives could indicate that this tissue is already somewhat biased to its normal fates (epidermal/anterior neural plate) (Fig. 1 *H* and *I*) (4, 11, 29, 30) by stages HH3+/4. In light of this, the finding that both types of graft generate cells with resident behavior and node identity is even more striking.

### Prospective Node Cells Are Plastic.

To test whether normal node precursor cells are committed to node and axial identities before entering the node, we prevented lateral epiblast cells from being recruited into the node by grafting them to a remote anterior position (Fig. 1*J*). After culture to HH8-10, graft-derived cells localize to and resemble head structures (such as the anterior neural plate and head epidermis, the latter pictured in Fig. 1*K*) rather than node-derived tissues and also fail to express the node marker, Chordin (*SI Appendix*, Fig. S2). Lateral cells therefore develop according to their new anterior position (4, 11, 29, 30) (Fig. 1 *H* and *I*), demonstrating that cells normally destined to give rise to node and axial identities are not committed to these before they enter the node.

### The Node Specifies Self-Renewal.

We then asked whether resident cells specified by the node are stem cells by testing for self-renewal, a key characteristic of a stem cell (26–28). First, the anterior epiblast was made to enter the node by grafting adjacent to it at HH3+/4 (*SI Appendix*, Fig. S3 *A* and *B*). Following culture to HH8-10, 2 to 10 GFP^+^ cells remaining in the node were regrafted into a second, younger (HH3+/4) host node (*SI Appendix*, Fig. S3 *C*–*F*), to determine whether the GFP^+^ cells can self-renew and contribute daughters to the developing axis for a second time. GFP^+^ cells contributed to both node and axis in 4 of 23 embryos (*SI Appendix*, Fig. S3 *G*–*I*), suggesting that resident cells specified by the node can indeed self-renew.

**Fig. 2.**
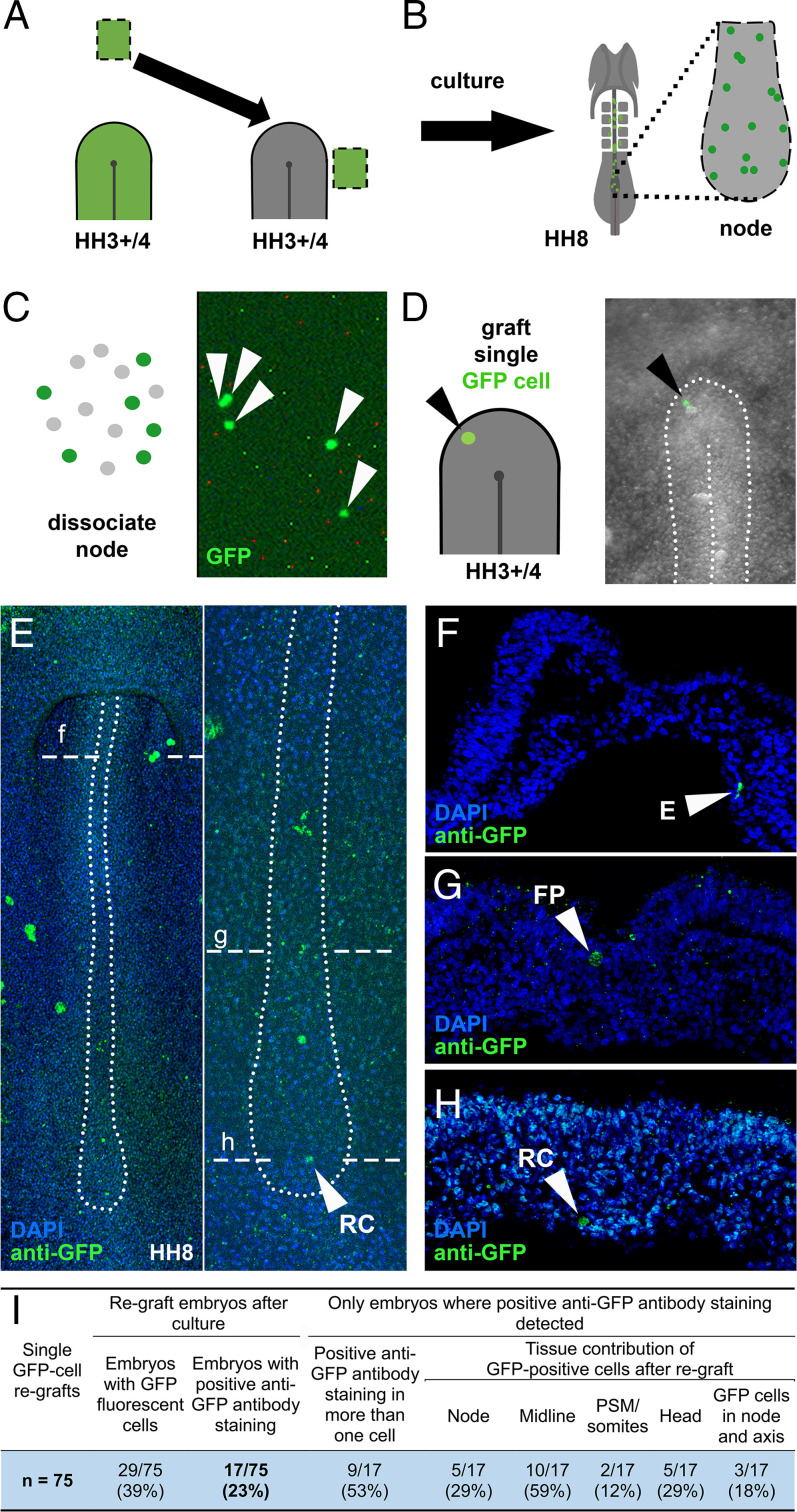
Single-cell regrafts reveal that the node can specify self-renewing resident cells. (*A*) Anterior epiblast not normally fated to enter the node was made to do so. (*B* and *C*) After culture, cells still residing in the HH8 node (*B*) were dissociated (*C*). (*D*) A single GFP^+^ cell was isolated and grafted into a second younger, HH3+/4 host node. (*E*–*H*) After culture (*E*), the single GFP^+^ cell had proliferated and descendants were found resident in the node and along the axis (*F*–*H*). (*I*) Summary of all single GFP cell regrafts. Transverse dashed lines show level of sections. E, endoderm; FP, floorplate; RC, resident cell. The size of the field of view is 0.5 mm x 0.8 mm (*D*), 3.5 mm x 1 mm (*E*), and 0.2 mm x 0.3 mm for the sections (*F*–*H*).

To assess cell division definitively and confirm self-renewal at the single-cell level, we repeated this regraft experiment using single GFP^+^ cells. Once again, two successive hosts were used, the first to specify resident cells from nonnode cells, and the second to test whether one of these resident cells could then continue to self-renew and contribute to axial structures when challenged to do so in a younger host (Fig. 2). This method would allow us to follow behavior of an individual GFP cell of known history across two hosts. After culture of the second host to HH8-10, anti-GFP staining revealed GFP^+^ cells in 17 of 75 grafted embryos, of which 9 had multiple GFP^+^ cells, showing that cell division had occurred (Fig. 2*I*). Three embryos had GFP^+^ cells in both node and axis, revealing that the GFP resident cells both self-renewed and contributed progeny to the axis of the second host (Fig. 2 *E*–*H* and *SI Appendix*, Fig. S3 *J*–*M*). The ability of the node to specify self-renewing resident behavior from cells not normally destined to enter it, even after challenging the cell by a heterochronic transplant, is fully consistent with the properties of an instructive niche (23–25).

### Locating Long-Term Resident Cells.

**Fig. 3.**
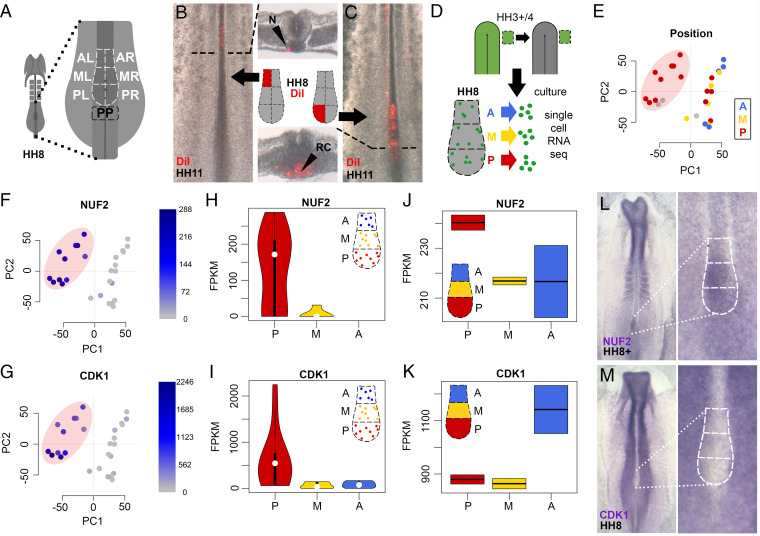
Long-term resident cells reside in the posterior node and are enriched in G2/M-phase cell-cycle genes. (*A*–*C*) Fate mapping of six domains reveals that only posterior subregions continue to contribute to resident cells. (*D*) Individual resident cells originating from lateral epiblast were isolated from anterior (blue), middle (yellow), and posterior (red) HH8 node subregions and processed for scRNA-seq (dataset comprises 27 cells). (*E*–*I*) PC analysis reveals a cluster of posterior cells (pink oval) (*E*) enriched in G2/M-phase-related genes, including NUF2 (*F*–*H*) and CDK1 (*G*–*I*) (intensity of blue reflects FPKM levels). Gray dots represent cells whose position in the node could not be determined at the time of collection. (*J*–*M*) Bulk RNA-seq (*J* and *K*) and in situ analysis (*L* and *M*) reveals that while some cell-cycle genes enriched in posterior resident cells are also enriched in the posterior node as a whole (e.g., NUF2, *J* and *L*), others are enriched only in a subset of cells from the posterior node with greater expression in the anterior node as a whole (e.g., CDK1, *K* and *M*). Field of view is 1 mm x 2.5 mm (*B* and *C*) and 2.5 mm x 5 mm (*L* and *M*).

Does the entire node act as a niche, or is this property located in a particular subregion? To identify the regions containing long-term resident cells and thus the most likely locations for the niche, we constructed a fate map of the node by labeling each of six subregions using a lipophilic dye (DiI) at HH8 (Fig. 3*A*). After culture to HH11-12, all cells from anterior subregions had come out from the node, while cells arising from the middle subregions contributed to anterior parts of the later node (chordoneural hinge). Only the posterior subregions continued to contribute to the entire older node and its derivatives (Fig. 3 *B* and *C* and *SI Appendix*, Fig. S4). No substantial difference to axial contributions was found for equivalent left and right node regions (*SI Appendix*, Fig. S4*G*). Collectively, this suggests that resident cells remaining in the node the longest are confined to posterior subregions. Consistent with this, in the single-cell regrafting experiments described earlier, when GFP cells were observed in both the node and the axis, there was always a GFP cell in the posterior node subregion (RC in Fig. 2*E* and *SI Appendix*, Fig. S3 *J* and *L*). Within the posterior subregion, these resident cells were observed in both dorsal (*SI Appendix*, Fig. S3*K*) and ventral (Fig. 2*H*) positions. Live imaging of embryos, in which a mosaic of cells was fluorescently labeled, also revealed endogenous resident cells remaining in the posterior node as it regresses (HH5-9), whereas most cells from other regions of the node were left behind to contribute to the axis (Movie S1 and stills from the movie in *SI Appendix*, Fig. S5). The posterior node is therefore the most likely region to contain an axial stem cell niche.

### Molecular Properties of Resident Cells.

What are the molecular characteristics of cells residing in this posterior niche? We grafted GFP-epiblast from next to the node to the same position in a wild-type host and cultured the embryos to HH8. Single graft-derived cells were collected from the posterior, middle, and anterior regions of the HH8 node, and processed for scRNA-seq using SmartSeq (Fig. 3*D*). The data were examined by principal component analysis (PCA). We found that the first two components explain the greatest proportion of variance (*SI Appendix*, Fig. S6*A*), with the first component (PC1) grouping cells into two clusters, one composed largely of cells collected from the posterior part of the node (Fig. 3*E*). To identify the genes causing this clustering, we calculated the correlation coefficient of genes with PC1. Thirty-seven genes have significant expression in the posterior cluster (correlation coefficient < −0.8); strikingly, the majority of these (31 of 37) encode proteins of the G2/M phases of the cell cycle (*SI Appendix*, Fig. S7), including NUF2 and CDK1 (Fig. 3 *F* and *G*). This suggests that these cells in the posterior node are preparing to divide. Graft-derived cells isolated from other parts of the node appear to be randomly distributed in other phases of the cell cycle (*SI Appendix*, Fig. S8).

This finding raises the question of whether the posterior region of the node is a unique site where cells pass through the G2/M phases of the cycle. Spatial analysis of dividing cells using pH3 staining in the regressing node reveals dividing cells in anterior, middle, and posterior regions of the node, with no evident accumulation in the posterior subregion (*SI Appendix*, Fig. S9 *A*–*D*). To explore this further, we performed spatial transcriptomics by comparing anterior, middle and posterior node subregions at HH8. Half (16 of 31) of the cell-cycle related genes identified in the scRNA-seq, including NUF2, were also enriched in the posterior node as a whole (Fig. 3 *H* vs. *J* and *SI Appendix*, Fig. S9), while the remaining 15, including CDK1, were instead enriched in anterior node regions (Fig. 3 *I* vs. *K* and *SI Appendix*, Fig. S9). In situ hybridization for these genes confirmed the mRNA distribution suggested by RNA-seq of entire node subregions (Fig. 3 *J*–*M*). These results show that among the node cells originating from the lateral epiblast, enrichment of G2/M-phase cell cycle genes is restricted to resident cells in the posterior node subregion, but that this is not necessarily true for all cells of the node. This observation is also consistent with the idea, from previous studies, that resident cells may only represent a small proportion of cells in the node and thus the transcriptomic signature of entire node regions is not equivalent to that of individual resident cells within such regions. Interestingly, the transcriptomes of entire subregions reveal an enrichment of genes involved in Wnt, Notch, and FGF signaling in the posterior part of the node (*SI Appendix*, Fig. S10), three pathways that have been implicated in stem cell niches (25).

### Competence of Epiblast Cells to Respond to the Node.

**Fig. 4.**
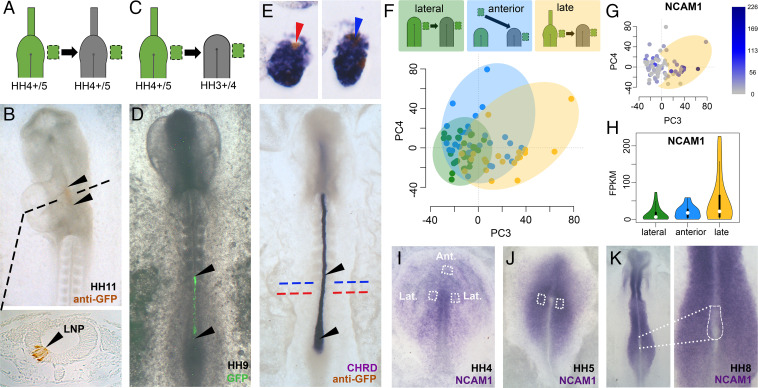
Older epiblast is not competent to respond to the node. (*A* and *B*) (“Late”) Epiblast lateral to the HH4+/5 node normally contributes to lateral neural plate (LNP). Transverse dashed line shows level of accompanying section. (*C*–*E*) When made to enter the younger node (*C*), late epiblast contributes to the axial midline (*D* and *E*). Black arrowheads show the extent of head-to-tail contribution. While some graft-derived cells in the notochord express the notochord marker CHRD (blue arrow), others do not (red arrow). (*F*–*H*) Single cells from the HH8 node plotted using PC3/4 (dataset comprises 77 cells) cluster according to their epiblast origin (lateral, anterior, or late) (*F*). Expression of neural plate-like genes, including NCAM1, correlates with late cells (*H*) that largely do not overlap with the lateral graft derived cluster (*G*). Yellow oval in *G* shows late graft-derived cells that do not overlap with lateral graft-derived cells. Intensity of blue in *G* reflects FPKM levels. (*I*–*K*) In situ hybridization reveals higher levels of NCAM1 expression at the time of grafting in late (*J*) versus early (*I*) stage donor grafts and a neural plate distribution of NCAM1 (*J* and *K*). Ant., anterior epiblast; Lat., lateral epiblast. Field of view is approximately 4 mm x 2.5 mm (*B*), 6 mm x 2.5 mm (*D* and *E*), 5 mm x 3.5 mm (*I* and *J*), and 5 mm x 2.5 mm (*K*, low magnification image).

Is there a limit to the period during which lateral epiblast cells can respond to the node environment (their “competence” to respond to node signals that instruct them to become resident)? To answer this, lateral epiblast from older-stage embryos (HH4+/5, corresponding to the prospective neural plate, Fig. 4 *A* and *B*) was used as donor tissue (5, 8). This HH4+/5 epiblast was forced to enter a younger (HH3+/4) node by grafting just adjacent to it (Fig. 4 *C*–*E*). After culture to HH8-11, graft-derived cells from these late-to-early transplants contributed to the same axial and paraxial structures as the grafts from younger donors described earlier (*SI Appendix*, Fig. S11*A*). However, late epiblast gave rise to cells with resident behavior in fewer embryos (55%, *n* = 6 of 11) than the lateral epiblast (89%, *n* = 8 of 9), suggesting that late epiblast cells are less able to respond to the node environment. Late epiblast cells also contribute to mesodermal structures (notochord and PSM/medial somite) less frequently than lateral epiblast and some of those that do end up in these tissues fail to express appropriate mesodermal genes (Fig. 4*E*). In contrast, late grafts contribute more frequently to neural structures (floorplate, 45% and lateral neural plate, 27%) than early lateral epiblast (33% and 11%, respectively) (*SI Appendix*, Fig. S11*A*).

At the time of excision from the late donor (HH4+/5), lateral epiblast cells already express neural plate markers (including ZEB2 and SOX2) (*SI Appendix*, Fig. S11 *C* and *F*), but during their subsequent development in the host they lose expression of these genes, except for descendants that become located in neural structures (*SI Appendix*, Fig. S11 *H*–*K*). This suggests that the node environment causes late epiblast cells to lose their neural plate identity, but is not sufficient to convert them fully into axial mesoderm. This transition in bias away from mesodermal and toward neural fates appears to take place around stages HH5− to HH5 (*SI Appendix*, Fig. S11*L*), which corresponds to just a couple of hours after epiblast lateral to the node normally stops entering the node (5, 8). Interestingly, the contribution of anterior epiblast from younger (HH3+/4) donors to neural and mesodermal structures was similar to that of late epiblast (*SI Appendix*, Fig. S11*A*). Since the normal fate of this anterior epiblast is neural/epidermal, and the normal fate of late epiblast used is neural plate, this suggests that a bias toward their normal neural fates is present even at the earlier stage. Together, these results separate the competence of epiblast cells to be directed to a mesodermal fate from their competence to be recruited as resident cells and contribute to the elongating axis in response to the node environment.

What molecular changes accompany this change in competence? To investigate this, we performed scRNA-seq on cells with resident behavior at HH8 originating from late-to-early and anterior grafts and compared these to fully competent cells (derived from grafts of early lateral epiblast) (*SI Appendix*, Fig. S12 *A*–*C*). This dataset therefore provides us with transcriptomes of cells with known origin (late or early lateral or anterior epiblast), movements (passing into the node from adjacent epiblast) and position within the HH8 node at the time of collection (anterior, middle, or posterior). The data were examined by PCA and using U-maps (*SI Appendix*, Fig. S12). We found that the first three components explain the greatest proportion of variance (*SI Appendix*, Fig. S12*D*).

Irrespective of origin, the most significant variation among the cells can still be accounted for by their expression of G2/M-phase related cell-cycle genes (*SI Appendix*, Fig. S12 *F*–*H*, compare with Fig. 3 *F* and *G*; clustering by PC1-2), although clustering of posterior resident cells is no longer as clear as when the lateral epiblast is assessed alone (*SI Appendix*, Fig. S12*E*, compare with Fig. 3*E*; clustering by PC1-2). Some host cells obtained from the same node regions have a similar signature, revealing that this is not an artifact of grafting (*SI Appendix*, Fig. S12 *O*–*Q*). However, PC3/4 cluster cells into overlapping groups according to their donor origin (early lateral, anterior, or late lateral epiblast) (Fig. 4*F* and *SI Appendix*, Fig. S13). Cells originating from the early lateral epiblast form a fairly compact cluster, with cells from the late and anterior grafts partially overlapping and spreading along PC3 and PC4, respectively. Cells not overlapping with the cluster of lateral graft-derived cells are associated with reduced response to the node (i.e., they become resident but not all of them acquire a node molecular signature).

To identify genes associated with cells with such a reduced response, we analyzed PC3, which uncovers this cluster, revealing expression of 15 genes (correlation coefficient > 0.55 with PC3) (*SI Appendix*, Fig. S14*A*). Of these genes, seven have known roles in cell adhesion or in neural development, including NCAM1 and CLDN1, normally expressed mainly in the neural plate, present in lower levels in early (HH3+/4) epiblast and largely absent from the node (Fig. 4 *G*–*K* and *SI Appendix*, Fig. S14 *B*–*F*). This is consistent with the idea that these later cells, originating from the prospective neural plate, have already initiated their differentiation into the neural plate. These experiments therefore separate the responsiveness of epiblast cells to be recruited as resident cells by the node environment from their ability to acquire the identity of a different germ layer.

## Discussion

Apart from a few studies using prospective single-cell fate mapping (10, 16, 17), evidence for resident cells in the node has been based on studies of cell populations (14, 15, 18–20). Without challenging behavior at the single-cell level, it is not possible to test whether the node represents an instructive stem cell niche, where “instructive” signifies that it can impart resident, self-renewing properties even to cells that would not normally do so in normal development (31). Here, using single-cell grafts, we report that the node can confer resident and self-renewing behavior to epiblast cells that would not normally enter this domain. Cell dynamics and fate mapping locate the most likely site for such a niche to the caudal-most region of the node, which is consistent with fate-mapping results of the mouse node, showing that some labeled cells remain in the node–primitive-streak border as the axis forms (20, 32). In addition, in mouse, when the embryonic day (E)8.5 anterior node (which does not normally contribute to resident cells) is grafted to the node–streak border (caudal-most node), the grafted cells contribute to the chordoneural hinge and tailbud mesoderm (20), while E8.5 caudal lateral epiblast grafted to the node–streak border results in increased contribution of graft-derived cells to the chordoneural hinge (33). These results in mouse support a role for the caudal-most node in imparting resident behavior on cells, although, since groups of cells were grafted, this does not provide information about self-renewal. If the posterior part of the node is removed in either chick or mouse, axial elongation is impaired (15, 32), further illustrating the importance of the posterior node in axial development. The posterior node is therefore the strongest candidate for an axial stem cell niche in amniotes.

Whether or not such a niche might contain support cells that define or maintain it is yet to be determined. We have attempted to investigate the influence of neighboring cells on a cell’s resident behavior by comparing the outcome of grafting a single GFP cell into the node alone, or along with its non-GFP neighbors. We observed a small increase in the contribution of GFP progeny to the node from grafts containing non-GFP neighbors (two of nine and three of eight, respectively) (*SI Appendix*, Fig. S15). Although the number of cases is too low to draw statistically significant conclusions, this result raises the possibility that resident behavior might be influenced by neighboring support cells and therefore warrants further investigation.

Within the posterior node, single-cell grafts show that self-renewing resident cells can be found in both dorsal and ventral locations. In mouse, it has been suggested, based on BrdU staining, that cells in the dorsal part of the node are fast-cycling, while those of the ventral node are largely quiescent, with the exception of the caudal-most part of the node (32, 34). Although dorsoventral distribution of dividing cells in chick is not as well defined, the presence of self-renewing resident cells in both ventral and dorsal parts of the posterior node is consistent with the distribution of actively dividing cells in mouse.

The finding from scRNA-seq that resident cells from the posterior part of the node are enriched in G2/M-related cell-cycle genes is intriguing. There are several possible interpretations. It could suggest that the G2/M phases of the cell cycle are particularly long in this region of the node or particularly short in more anterior regions of the node. Another possibility is that cells in the node move around the node according to their position in the cell cycle, moving to the posterior node subregions during the G2 and M phases, although such cell movements have not been observed in live imaging of node cells and therefore this is unlikely to be the dominant pattern of behavior. However, a region in the node where cells preferentially divide is consistent with the idea of “transition zones” within the node, proposed by Mathis et al. (13). In this model, cells located posteriorly move little relative to one another, while those in more anterior regions disperse more before eventually leaving the node (13). Thus, if cell division of resident cells occurs mainly posteriorly, increased dispersal through anterior regions may result in their daughters reaching G2/M phases only after they leave the node anteriorly (notochord) or laterally (presomitic mesoderm), resulting in fewer dividing cells in the anterior regions of the node. In support of this, cell division of notochord progenitors in the node has previously been estimated to be 3.5 to 4 h (10, 21), which is compatible with the time taken by daughters to transition through the node and leave anteriorly. In the present study it was impossible to calculate the length of the cell-division cycle for resident cells, or the rate of cell death or any changes in cell division rate between progenitors and their descendants. However, localized cell division could account for previous suggestions that cells entering the neighboring presomitic mesoderm from the node do so at a specific stage of the cell cycle (35, 36).

Despite these arguments, the results of pH3 staining show dividing cells in the anterior, middle, and posterior regions of the node, with no clear accumulation in just one subregion, suggesting that although localized cell division may occur in a subset of cells, it is not the dominant behavior of cells in the node. It is important to note that the scRNA-seq experiment in the present study was designed to analyze a specific population of resident cells in the node, which originates from the epiblast lateral to the node and enters the node during gastrulation. In addition to cells recruited from the epiblast adjacent to the tip of the primitive streak, the node is also derived from an early-arising population of cells that accompany the tip of the streak as it elongates during gastrulation (2–4), as well as cells recruited from more posterior streak during node regression (*SI Appendix*, Fig. S4 *D* and *E*). Cells that pass through the node without becoming resident may divide in any region of the node, this being the dominant behavior observed when examining the entire node. Therefore, by targeting only a subset of cells contributing to the node, we are revealing a specific population of resident cells in the node that resides and divides in the posterior part of the node, which, due to its small size, goes largely unnoticed when cell division is assessed across all cells. The small size of this resident cell population agrees with previous suggestions that the node contains only a few self-renewing cells: based on cell cycle length and number of cells per somite, it was estimated that the number of progenitors for the medial half of the presomitic mesoderm (and the resulting somites) could be as few as 64 in the HH4 node (which comprises around 1,000 cells) (10, 21, 37).

We have shown that the node can instruct cells to become resident and self-renewing at least up to HH8, and it is possible that this property persists much later, even to tailbud stages. No prospective single-cell lineage tracing has yet been done for the full duration of axial elongation mainly due to technical limitations. However, analysis of retrospective single-cell labeling in mouse suggests that the population of resident progenitors may change over time, with some loss and addition of cells occurring between regressing node and tailbud stages (38). It has been proposed that some of these new cells might come from caudal lateral epiblast (39), even though the bulk of the epiblast lateral to the node stops ingressing into the node at a much earlier stage (HH4+/5−) (5, 8) as it loses the ability to acquire expression of node and mesoderm markers (present study).

In conclusion, in addition to its well-known roles as an “organizer” of the amniote embryo (40–42) and its ability to dorsalize mesoderm (43, 44), we provide evidence that the amniote node can also function as a stem cell niche that can specify cells to become resident and to self-renew to contribute to the elongating head–tail axis.

## Materials and Methods

### Embryos.

Wild-type chicken embryos were obtained from Brown Bovan Gold hens (Henry Stewart Farm) and from Rhode Island Red hens (Sunstate Ranch). Transgenic cytoplasmic GFP chicken embryos were supplied by the avian transgenic facility at The Roslin Institute, Edinburgh, and from Clemson University though Susan Chapman (14). All eggs were incubated at 38 °C in humidified incubators and staged according to Hamburger and Hamilton (1). For grafting experiments, ex ovo embryo cultures were prepared using the New technique (45) with modifications as described by Stern and Ireland (46).

### Epiblast Grafts.

Donor embryos were isolated in Tyrode’s solution (47). The donor embryo was turned ventral side up, underlying endodermal and mesodermal layers were peeled away and a piece of epiblast (∼20 to 50 cells) was cut out using 30-G syringe needles. Each epiblast piece was checked to ensure that no mesodermal/endodermal cells remained attached before grafting, and several “time = 0” sections taken to verify this (*SI Appendix*, Fig. S1 *B* and *C*), but we cannot entirely rule out the possibility that a few mesodermal/endodermal cells may have remained attached. An equal-sized piece of epiblast was removed from the host in the desired location and replaced with the donor epiblast. For “lateral-to-lateral” grafts (Fig. 1*D*), epiblast was grafted into the equivalent position in the host as its donor origin (i.e., “left-to-left” or “right-to-right”). The “lateral epiblast” was taken from immediately adjacent to the tip of the streak/node. The “anterior” epiblast was taken from a midline position, about half-way between the tip of the streak/node and the anterior area opaca.

For most grafts, the donor cells were from transgenic GFP embryos. To obtain nongrafted host node cells for scRNA-seq, a transgenic GFP embryo was used as the host and a non-GFP (wild-type) embryo as the donor. Lateral-to-lateral grafts were performed (as described above) using non-GFP wild-type tissue into a GFP host. After culture, only GFP cells (and not wild-type) were collected from the regressing node for scRNA-seq, to ensure that only host node cells were taken. All cells sequenced in this study therefore came from transgenic GFP embryos.

### Regrafts of Groups of Cells.

The first graft was an epiblast graft from a GFP donor to a non-GFP host (*SI Appendix*, Fig. S3 *A* and *B*). The second graft (regraft) included a group of cells from the first host’s node, containing 2 to 10 GFP^+^ cells alongside some neighboring GFP^−^ cells. A small “nick” was made in the node (ventral side) of the second host into which this group of cells was inserted using 30-G syringe needles to carefully maneuver the small pieces of tissue (*SI Appendix*, Fig. S3 *C*–*F*). This small injury heals very quickly (within minutes, sometimes even while the embryo is still under the microscope) without leaving any morphological trace. The grafted embryos were left at room temperature for ∼15 min to aid attachment of the graft to the host before further incubation. Embryos were cultured to HH8-10.

### Single-Cell Regrafts.

The first host had an “anterior-to-lateral” graft (Fig. 2*A*). After culture to HH8-10, a single GFP^+^ cell was collected from the host node (see *Single-Cell Manipulation*, below) and then transferred using a micropipette made from a pulled 50-µL calibrated micropipette (Drummond Scientific, Cat 2-000-050) attached to an aspirator tube, into the second host (HH3+/4) (Fig. 2 *B*–*D*). For some regrafts, a single GFP^+^ cell was transferred attached to one or more neighboring GFP^−^ cells from the first host (but there was never more than one GFP^+^ cell). A small “nick” was made in the node of the second host. The GFP^+^ cell was maneuvered into this nick by gently “blowing” saline on the cell with a micropipette. Ideally, once placed into its pocket, a flap of tissue would be used to cover the transplant site. The grafted embryo was then left at room temperature for ∼15 min to aid attachment of the cell to the host. Each New (45) culture was checked by fluorescence microscopy again just prior to incubation to ensure that the grafted GFP^+^ cell was still in place. Embryos were cultured to HH8-10.

### DiI Labeling.

The lipophilic dye, DiI (DiI-CellTracker CM, Molecular Probes Life Technologies, # C7001) was used for fate mapping of the HH8 node. Six separate subregions of the node were mapped: left and right sides of the anterior, middle, and posterior regions of the node (each subregion equal in length along the rostrocaudal axis) (*SI Appendix*, Fig. S4*A*). For 10 µL of working solution, 8.5 µL of 0.3 M sucrose and 1 µL of 1:20,000 Tween-20 were used with 0.5 µL of 2 mM DiI (in dimethylformamide). All components were first preheated at 65 °C, thoroughly mixed, and dissolved. The protocol for preparation and application of DiI was adapted from refs. 6, 10, and 48. The embryo was first prepared for New culture (45) and kept submerged in Tyrode’s. The node subregion to be labeled was cut out using 30-G syringe needles and transferred to a drop of Tyrode’s containing DiI (∼9:1 Tyrode’s: DiI working solution) and kept in the dark for 1 to 2 min. The tissue piece was then removed and washed in successive drops of Tyrode’s to remove any excess DiI before verifying that sufficient labeling had taken place, by fluorescence microscopy. The tissue piece was replaced into its original position, preserving the original dorsoventral orientation. Labeled embryos were cultured to ∼HH11-12. After culture, embryos were fixed in 4% paraformaldehyde in PBS for at least 4 d at 4 °C. To assess the location of the descendants of the DiI-labeled cells, several thick transverse sections were cut from each embryo by hand, using a scalpel, with the embryo pinned securely using insect pins in a silicon rubber-bottomed dish (47).

### In Situ Hybridization.

In situ hybridization with digoxigenin (DIG-)-labeled riboprobes was carried out following established protocols (3, 49, 50). Antisense DIG-riboprobes were synthesized by restriction digest and in vitro transcription. Plasmids used: CHRD (51), FOXA2 (52, 53); PARAXIS/TCF-15 (54); ZEB2 (8); SOX2 (55); TBX6 (56); DLL1 (57); RSPO3 (ChEST784h18); NKAIN4 (ChEST110n2); DRAXIN (ChEST545l1); CLDN1 (ChEST168n2); NCAM1 (ChEST845i20); MSGN1 (ChEST90p23); CHST15 (ChEST391h17); AKAP12 (ChEST376j15); FOXM1 (ChEST313o15); TOP2A (ChEST849a2); NUF2 (ChEST450j22); CENPL (ChEST97i12); MAD2L1BP (ChEST365n5).

### Immunohistochemistry.

Anti-GFP antibody staining largely followed the methods described by Stern (49) and Streit and Stern (50). Embryos were processed for anti-GFP antibody staining either immediately after collection (and fixing in 4% PFA overnight at 4 °C or at room temperature for 20 min) or following in situ hybridization. Embryos stained either with rabbit anti-GFP primary (Life Technologies, 1:2,000 dilution) followed by an HRP-conjugated secondary goat anti-rabbit IgG (Santa Cruz, 1:2,000 dilution) or with a goat anti-GFP primary antibody (Rockland, #600–101-215; 1:500 dilution) followed by donkey anti-goat Alexa Fluor 488 secondary antibody (1:1,000 dilution). HRP was revealed using DAB (Sigma, #D5637). pH3 was detected using mouse anti-histone H3, phospho S10 IgG1 (Abcam-ab443110, 1:500 dilution) followed by a donkey anti-mouse IgG Alexa Fluor 647 (Invitrogen, #A31571, 1:1,000 dilution).

### Histology.

Some embryos processed for in situ hybridization and anti-GFP antibody staining were embedded in paraffin wax and sectioned using a microtome. Methods largely followed those of Izpisúa-Belmonte et al. (3). All sections were transverse and 10-µM thick. Slides were mounted using a 3:1 solution of Canada balsam (Merck, #1016910100) and Histoclear (HS-202 HISTO-CLEAR II, National Diagnostics). Embryos stained with DAPI and anti-GFP antibody were embedded in gelatin and cryosectioned. Sections were 12-µM thick and slides were mounted using fluoromount (SouthernBiotech, #0100-01).

### Microphotography.

Images of all whole-mount embryos and thick sections were recorded using transmitted light with an Olympus SZH10 stereomicroscope with epifluorescence optics. Paraffin sections were examined on an Olympus Vanox-T optical microscope. A QImaging Retiga 2000R Fast 1394 camera and QCapture Pro software was used for image capture. DAPI and anti-GFP antibody stained embryos (whole-mount and sections) were imaged on a Zeiss Imager M2 with an ApoTome module.

### Live Imaging and Cell Tracking.

Electroporation mixture containing 1 mg⋅mL^−1^ pDsRed-Express plasmid, 6% (wt/vol) sucrose and 0.04% (wt/vol) Fast Green FCF was applied dorsally, just lateral to the node of HH4− embryos to transfect ingressing cells. Electroporation was performed in a custom-made chamber with four pulses of 5 V, 50-ms width, 500-ms interval. Embryos were then cultured using a modification of New’s method (45, 46) in 35-mm plastic dishes with a glass coverslip base, and imaged with a Zeiss LSM 880 inverted microscope using a Plan-Apochromat 20×, 0.8 NA objective. Images were acquired at 10-min intervals using 3 × 5 tiling (10% overlap) to achieve coverage of the whole embryo. Image analysis and cell tracking were performed using Imaris (Bitplane) software. The embryo was imaged from the epiblast (dorsal) side but the output from Imaris is displayed as a mirror image (pseudoventral view).

### Single-Cell Manipulation.

For collection of single cells for single-cell regrafts and scRNA-seq, the cultured embryo was first submerged in Tyrode’s solution (for regrafts) or sterile molecular grade PBS with 0.1% glucose (for scRNA-seq). The node was then divided into anterior, middle, and posterior subregions of equal rostrocaudal length. Each of these regions containing GFP^+^ cells was cut out, in turn, and placed in a drop of nonenzymatic dissociation medium (Sigma, # C5914-100ML), kept over ice. Each piece was washed twice in drops (∼30 µL) of this dissociation medium while over ice. To help with dissociation, after ∼5 min the tissue was gently aspirated up and down using a micropipette (made from a pulled 50-µL calibrated micropipette [Drummond Scientific, Cat 2-000-050] attached to an aspirator tube). The micropipette was broken at the tip to have a diameter just narrower than the width of the tissue piece. Once the piece of tissue was fragmented, a capillary with a narrower tip was used for further dissociation to single cells in suspension. GFP^+^ cells were identified by fluorescence under a dissection microscope (70× magnification) and were individually aspirated using a micropipette. The cell was transferred into a drop of Tyrode’s (for regrafts) or of sterile molecular grade PBS (for scRNA-seq) to replace the dissociation medium and to verify that there was only a single GFP^+^ cell. Once verified, the cell was transferred (using a fresh pulled micropipette) to the second host (for regrafts) or into a 200-µL tube containing 5 µL of lysis buffer and 5% RNase inhibitor (for scRNA-seq) (lysis buffer and RNase inhibitor from SMART-Seq v4 Ultra Low Input RNA Kit, Takara, # 634892). Once the dissociation process began, cells were collected for ∼20 min, after which time any remaining dissociated tissue was discarded, and a new tissue piece taken from the embryo.

### scRNA-Seq.

The SMART-Seq v4 Ultra Low Input RNA Kit (Takara, # 634892) targeting mRNA, was used for preparing the single cells for sequencing. Amplified cDNA was purified using AMPure magnetic purification beads (Agencourt AMPure XP, Beckman Coulter # A63880). The DNA concentration of purified cDNA was checked by Qubit (dsDNA HS Assay Kit, Thermofisher # Q33230). All samples yielding at least 5 ng of cDNA were sheared by sonication (Covaris, S220/E220 focused ultrasonicator, set to: 10% duty factor, 200 cycles per burst, 120-s treatment time, 175 W peak incident power) to obtain ∼500-bp fragments for library preparation. The ThruPLEX DNA-seq, Dual Index Kit (Takara, # R400406) was used to construct dual indexed libraries for each sample. Libraries were individually purified, using AMPure magnetic purification beads (Agencourt AMPure XP, Beckman Coulter # A63880). DNA concentration of purified libraries was checked by Qubit (dsDNA HS Assay Kit, Thermofisher # Q33230) and size distribution of cDNA measured using Tapestation (Agilent High sensitivity D1000 screen tape, #5067-5584). All libraries were individually diluted to 10 nM in elution buffer before pooling together. Pooled libraries were sequenced by University College London (UCL) Genomics using an Illumina NextSeq sequencer with a 75-bp single-end read cycle kit. The average number of reads per cell was ∼10 million (range: 6,838,400 to 15,404,851). The scRNA-seq raw data have been deposited in European Bioinformatics Institute (EBI) Array Express (accession nos. E-MTAB-9116 and E-MTAB-11216).

### RNA-Seq of Tissues.

Tissues (HH8 node subregions) were isolated from transgenic-GFP embryos using 30-G syringe needles in sterile molecular grade PBS. Six subregions (six samples) of the node were taken: anterior left, anterior right, middle left, middle right, posterior left, and posterior right. Tissues were collected into RNAlater (Invitrogen, #AM7020). For each sample (node subregion), tissues were collected from 13 to 17 embryos. RNA was extracted using the Micro Total RNA Isolation Kit (Invitrogen, #AM1931) and concentration and quality measured using Tapestation (Agilent High sensitivity RNA screen tape, #5067-5579). The NEBNext Single Cell/Low Input RNA Library Prep Kit for Illumina (# E6420) was used for cDNA and library synthesis (performed by UCL Genomics). Libraries were sequenced by Illumina NextSeq using a 75-bp single-end read cycle kit. The average number of reads per sample (node subregion) was ∼22 million (range: 19,572,310 to 24,523,360). The bulk RNA-seq raw data have been deposited in EBI Array Express (accession no. E-MTAB-9115).

### RNA-Seq Data Processing.

Raw data were checked using FastQC (58) to assess overall quality. Cutadapt (59) was used to remove low-quality bases (Phred quality score <20) at the 3′ and 5′ ends, adapter sequences, primer sequences, and poly-A tails of each read. Reads were aligned to the galGal6 chicken genome using TopHat2 (60), alignment rates were 91.9% ± 0.3% (for scRNA-seq) and 86.3% ± 0.65% (for RNA-seq of tissues). Transcripts were counted and normalized using Cufflinks (61) programs *cuffquant* and *cuffnorm*, respectively. Data analysis was performed in the R environment (R-3.5.1).

For scRNA-seq, all sequenced cells passed quality control. The matrix of transcript FPKMs (fragments per kilobase of transcript per million mapped reads) contains expression of 24,353 genes in 77 samples (cells). Of these, 13,817 are expressed (with an FPKM > 0.5) in at least two cells in our data. The top 5,000 most variable of these genes were used for PCA. PCA was carried out on data from single cells from the HH8 node originating only from lateral epiblast (*n* = 27) and from single cells from the HH8 node originating from anterior, lateral, and late epiblast (*n* = 77). In addition, we also analyzed single cells obtained from the host node (*n* = 15) to compare to the profile of cells from lateral epiblast-derived grafts. Correlation coefficients were calculated for the relationship between gene expression and a given PC.

For RNA-seq of tissues, all node subregions passed quality control. Mitochondrial RNAs, ribosomal RNAs, and microRNAs were excluded. FPKM values for left and right subregions for anterior, middle, and posterior were averaged (mean) to give a single FPKM value for each gene for anterior, middle, and posterior subregions. Fold-changes for each gene in the posterior subregion against its expression in middle and anterior subregions were calculated to find genes with the most marked differential expression between subregions (*SI Appendix*, Fig. S10*B*).

## Supplementary Material

Supplementary File

Supplementary File

## Data Availability

The scRNA-seq and bulk-RNA-seq raw datasets generated during the current study are available in the EBI Array Express, https://www.ebi.ac.uk/arrayexpress/ (accession nos. E-MTAB-9115, E-MTAB-9116, and E-MTAB-11216). All other study data are included in the main text and supporting information.
