# Supplementary material for: The embryonic node behaves as an instructive stem cell niche for axial elongation
Source: Proc Natl Acad Sci U S A. 2022 Jan 31;119(5):e2108935119. doi: 10.1073/pnas.2108935119 (PMC8812687; doi:10.1073/pnas.2108935119)
Supplement: Supplementary File [file pnas.2108935119.sapp.pdf]

PNAS
www.pnas.org

Supplementary Information for

The embryonic node behaves as an instructive stem cell niche for axial elongation

Authors: Tatiana Solovieva[1], Hui-Chun Lu[1], Adam Moverley[1, 2], Nicolas Plachta[2] and Claudio D. Stern[1]*

Affiliations:

[1] Department of Cell and Developmental Biology, University College London

[2] Institute of Molecular Cell Biology, A*STAR, Singapore

* Claudio D. Stern - Corresponding author

Email: c.stern@ucl.ac.uk

This PDF file includes:

Figures S1 to S15
Legend for Movie S1
Legends for Datasets S1 to S2
SI References

Other supplementary materials for this manuscript include the following:

Movie S1
Datasets S1 to S2

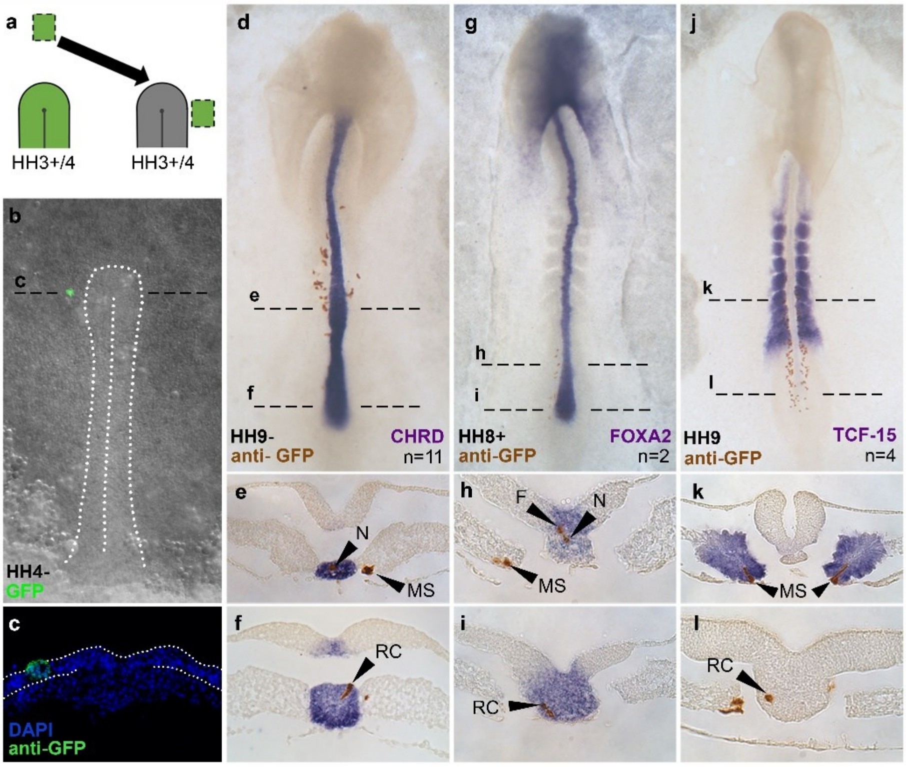

Fig. S1. Anterior epiblast derived cells made to enter the node express genes appropriate to their new location. a, Schematic of graft using anterior epiblast from a GFP donor. Embryo shortly after grafting anterior epiblast lateral to the node **(b)** with a section showing that the graft is in the epiblast only **(c)**. White dotted line in ‘c’ outlines the epiblast. Embryos grafted as in ‘a’ shown after culture to HH8-10 and processed for *in situ* hybridization for the node and notochord markers CHRD **(d-f)** and FOXA2 **(g-i)** and the somite marker TCF-15/paraxis **(j-l)** and stained for anti-GFP antibody (brown). All whole-mount embryos shown in ventral view. n-numbers indicate the number of embryos grafted. N, notochord; MS, medial somite; F, floorplate; RC, resident cell; CNH, chordoneural hinge; PSM = presomitic mesoderm.

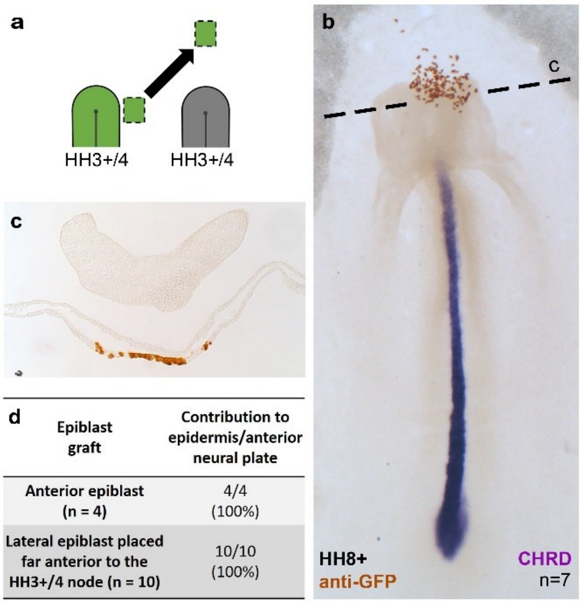

Epiblast graft	Contribution to epidermis/anterior neural plate
Anterior epiblast (n = 4)	4/4 (100%)
Lateral epiblast placed far anterior to the HH3+/4 node (n = 10)	10/10 (100%)

Fig. S2. Epiblast normally fated to enter the node does not give rise to cells with axial mesodermal identity if prevented from entering the node. a, Epiblast lateral to the node was prevented from entering the node by grafting it to a distant site, located much more anteriorly. **b-c,** After culture to HH8-9, lateral graft-derived cells (shown by anti-GFP) do not express Chordin (which is normally expressed in node and notochord). **d,** Table summarising the contribution of graft-derived cells to the axis, originating from anterior epiblast (example in Fig. 1h-i) or from lateral epiblast placed far anterior to the node (as in Fig. 1j-k and this figure, 'a-b').

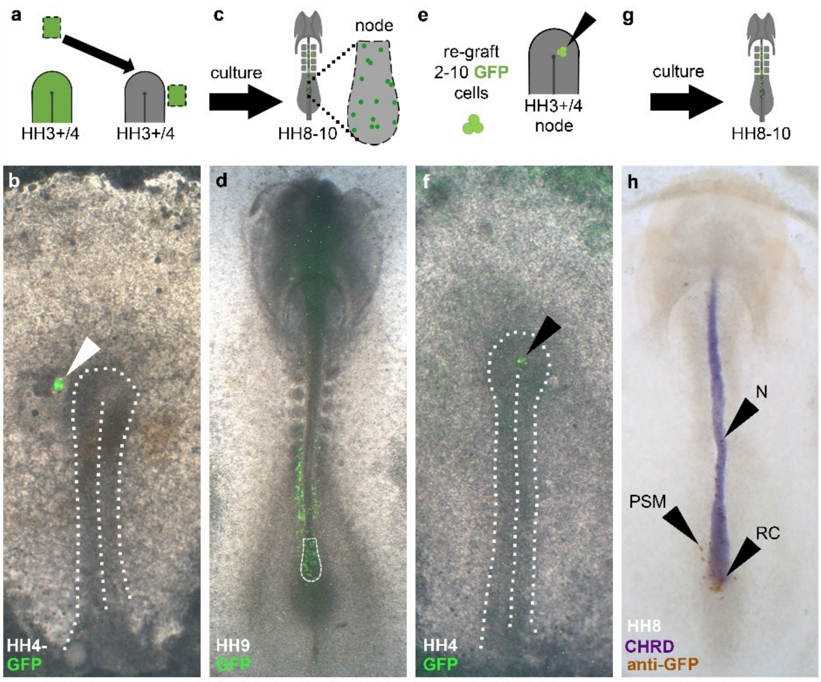

i

Re-graft of several GFP cells	Second host with GFP fluorescent cells after culture	Tissue contribution of GFP-positive cells in second host				
		Node	Midline	PSM/ somites	Head	GFP cells in node and axis
**n = 36**	**23/36 (64%)**	7/23 (30%)	9/23 (39%)	8/23 (35%)	2/23 (9%)	4/23 (17%)

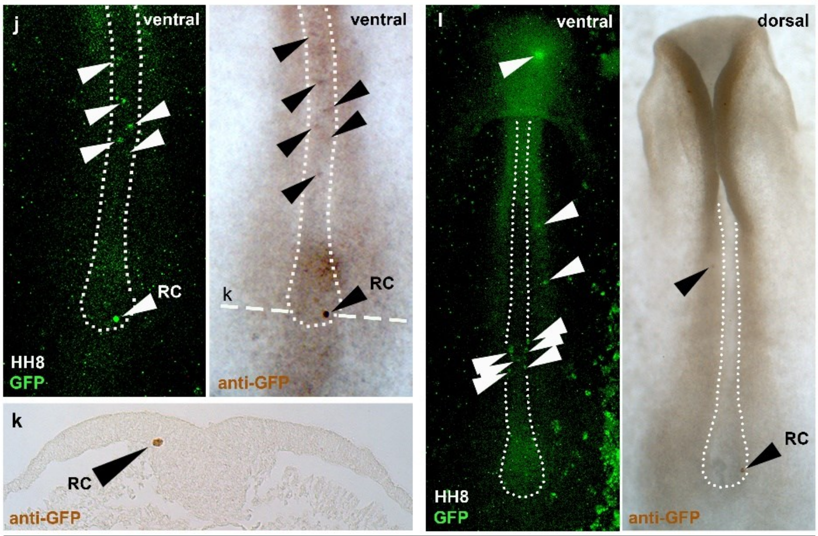

m

Single GFP-cell re-grafted embryos with multiple GFP-cells after culture (n=9)	Tissue contribution of GFP-cells after culture			
	Node	Midline	Somite	Head
embryo 1				
embryo 2				
embryo 3				
embryo 4				
embryo 5				
embryo 6				
embryo 7				
embryo 8				
embryo 9				

Fig. S3. Resident cells specified by the node can contribute to the node and axis for a second time. a-h, Re-grafts of groups of GFP-positive cells with resident behaviour. First, anterior epiblast was grafted just lateral to the HH3+/4 node (**a-b**). (White arrow = GFP-cells in host). Embryos were then cultured to HH8-10 (**c-d**). Two-to-ten GFP-positive cells were taken from the regressing node (outlined in 'd'), attached to some non-GFP neighbours and transferred to the node of a second, younger, HH3+/4 host (**e-f**). (Black arrows in e-f mark the re-grafted group of cells). After culture to HH8-10, GFP-positive cells can be found in both node and the midline (**g-h**). Black arrows in (h) point to some of the GFP-positive cells that contributed to notochord (N), presomitic mesoderm (PSM) and resident cells in the node (RC). (b) and (d) correspond to the same embryo; (f) and (h) are also two views of the same embryo. Cells re-grafted into the embryo in (f) were taken from the embryo shown in (d). All embryos shown in ventral view. **i,** Summary table showing tissue contributions to the second host from re-grafted groups of GFP-positive resident cells (as in h). **j-l,** Two embryos (j-k and l) following a single GFP cell re-graft (as in Fig 2e), showing distribution of GFP cell progeny along the axis after culture. Arrows show GFP-positive cells under fluorescence (white arrow) and after anti-GFP antibody staining (black arrows). RC, resident cell; N; notochord; PSM, presomitic mesoderm. **m,** Summary table showing tissue contributions of GFP-cells across individual embryos following single-cell GFP re-grafts for those embryos in which more than one GFP-cell was seen after culture (9 embryos).

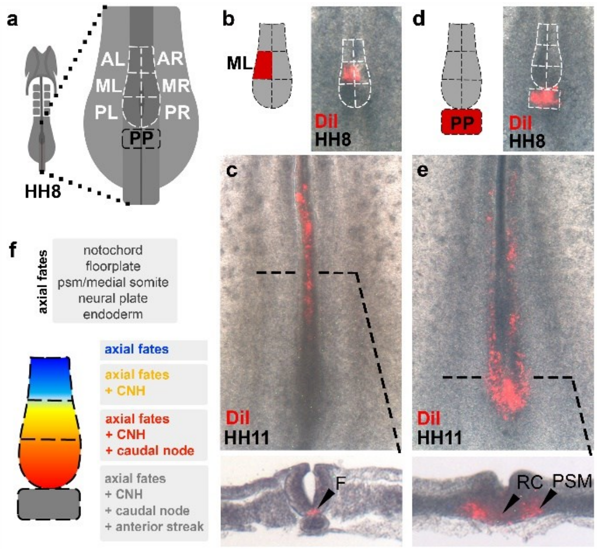

g

DiI-labelled region	Tissue contribution of DiI-labelled cells after culture (% of embryos)							
	Anterior streak	Caudal node	CNH	Noto-chord	Floor-plate	PSM/ medial somite	Neural plate	Endo-derm
AL (n = 3)	0	0	0	100	100	33	100	33
AR (n = 2)	0	0	0	100	100	50	0	50
ML (n = 5)	0	0	80	100	100	100	60	20
MR (n = 4)	0	0	50	100	100	75	50	25
PL (n = 4)	0	50	50	75	50	100	50	25
PR (n = 3)	0	100	100	100	100	100	0	33
PP (n = 3)	100	100	100	100	66	100	33	33

Fig. S4. DiI fate mapping of HH8 node/anterior streak sub-regions. HH8 node outlined with white-dashed line and sub-divided into six sub-regions (ventral view) (**a**): anterior left (AL), anterior right (AR), middle left (ML), middle right (MR), posterior left (PL), posterior right (PR), anterior streak just caudal to the posterior part of the node (PP). Example of labelling a middle sub-region (**b**) and resulting embryo after culture (**c**). Example of labelling the anterior streak (**d**) and resulting embryo after culture (**e**). Summary of node sub-region fates illustrated in (**f**) and shown in a table in (**g**). n-numbers refer to the number of embryos labelled. The intensity of the red background reflects the percentage of embryos with labelled cells in the region shown in each column. F, floorplate; PSM, presomitic mesoderm; RC, resident cell.

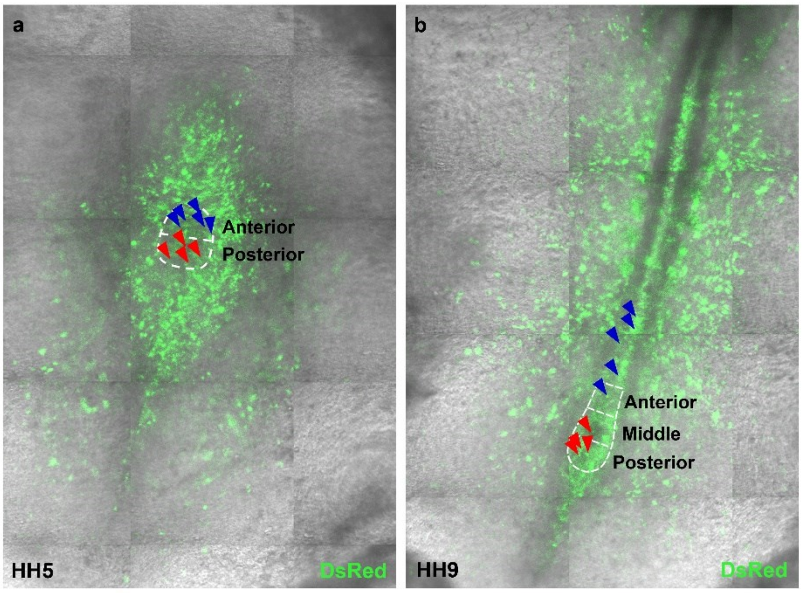

Fig. S5. Cell tracking reveals that cells in the posterior node at HH5 remain resident within the node while those in the anterior part leave the node to become dispersed along the notochord. Stills from Movie S1, showing a mosaic of cells labelled with DsRed (pseudo-color encoded as green) and the regressing node (dashed white outline) segmented and divided into anterior, middle and posterior sub-regions (dashed white lines). Cells from the anterior node (blue arrows) and posterior node (red arrows) were selected at HH5 **(a)** and then tracked through to HH9 **(b)**. See Movie S1.

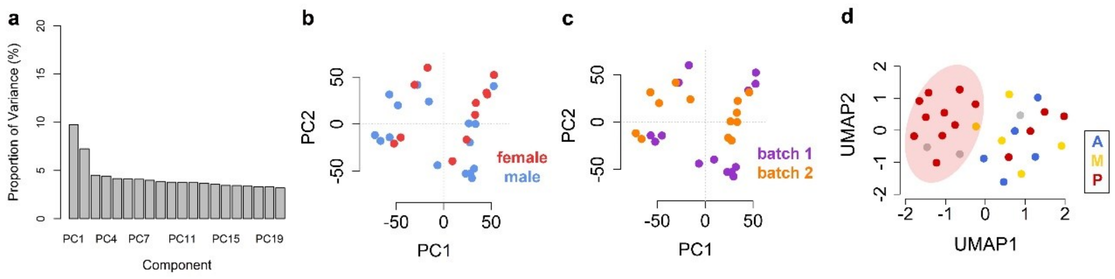

Fig. S6. Exploring variables defining cell clustering. The dataset comprises 27 cells collected from the HH8 node, originating from lateral epiblast grafts (as shown in Fig. 3d). The first 20 (out of 27) principal components are shown. The first two PCs provide the most informative measure for clustering (**a**). Cell gender (male/female) (**b)** and sequencing batch **(c)** did not account for clustering by either of the first two PCs. UMAP analysis of the 27 sequenced cells **(d)** reveals clustering comparable to PC1 versus PC2 analysis, with one cluster composed predominantly of posterior (red) cells (pink oval) (compare to Fig 3e). A, anterior; M, middle; P, posterior.

Gene	Correlation coefficient with PC1	Full gene name	Cell-cycle role
Not annotated	-0.92	ENSGALG00000033792	-
**TUBA1B**	-0.91	Tubulin Alpha 1b	-
**KNSTRN**	-0.91	Kinetochore-Localized Astrin-Binding Protein (SKAP)	Metaphase to anaphase progression (1)
**CENPH**	-0.91	Centromere protein H	Associated with the kinetochore complex (2, 3)
**BIRC5**	-0.90	Baculoviral IAP Repeat Containing 5 (survivin)	Metaphase to anaphase progression (4)
**NUF2**	-0.90	NDC80 kinetochore complex component	Associated with the kinetochore complex (5)
**CKS1B**	-0.89	CDC28 Protein Kinase Regulatory Subunit 1B	Initiates mitosis (6)
**HMGB2**	-0.89	High Mobility Group Box 2	-
**CDCA3**	-0.88	Cell Division Cycle Associated 3	Initiates mitosis (7)
**SKA3**	-0.88	Spindle And Kinetochore Associated Complex Subunit 3	Metaphase to anaphase progression (8, 9)
**PBK**	-0.88	PDZ Binding Kinase	Associated with the mitotic spindle (10, 11)
**HAUS1**	-0.87	HAUS Augmin Like Complex Subunit 1 (HEI-C)	Metaphase to anaphase progression (12, 13)
**HJURP**	-0.87	Holliday Junction Recognition Protein	Centromere chromatin assembly (14, 15)
**CENPO**	-0.87	Centromere Protein O	Associated with kinetochore complex (16)
**SMC2**	-0.87	Structural Maintenance Of Chromosomes 2	Component of both condensin complexes (17, 18)
**NCAPH2**	-0.87	Non-SMC Condensin II Complex Subunit H2 (CAPH2/ kleisin beta)	Component of the condensin II complex (19)
Not annotated	-0.86	ENSGALG00000048187	-
**KIF15**	-0.85	Kinesin Family Member 15 (HKLP2)	Associated with the mitotic spindle (20)
**MAD2L1BP**	-0.84	MAD2L1 Binding Protein (p31 comet, CMT2)	Metaphase to anaphase progression (21, 22)
**NCAPG**	-0.84	NON-SMC condensin 1 complex subunit G (CAP-G)	Component of the condensin I complex (23)
**CDK1**	-0.84	Cyclin dependent kinase 1	Initiates mitosis (24-26)
**PLK4**	-0.84	Polo kinase 4 (SAK)	Centriole duplication (27, 28)
**TUBA3E**	-0.84	Tubulin alpha 3E	-
**CENPC**	-0.84	Centromere protein C	Associated with the kinetochore complex (29, 30)
**FBXO39**	-0.84	F-Box Protein 39	-
**FOXM1**	-0.83	Forkhead box M1	De-represses late cell-cycle genes (31, 32)
**CENPU**	-0.83	Centromere Protein U	Associated with the kinetochore complex (16)
**TTK**	-0.82	TTK protein kinase (MPS1)	Metaphase to anaphase progression (33-36)
**KIF11**	-0.82	Kinesin Family Member 11 (EG5)	Associated with the mitotic spindle (37)
**KIF2C**	-0.82	Kinesin Family Member 2C (MCAK)	Associated with the mitotic spindle (38)
**CENPL**	-0.82	Centromere Protein L	Associated with the kinetochore complex (39)
**DEPDC1**	-0.82	DEP Domain Containing 1	Regulates mitotic progression (40)
**RACGAP1**	-0.82	Rac GTPase activating protein 1	Required for cytokinesis (41)
**TOP2A**	-0.81	Topoisomerase IIA	Alters DNA topology (42, 43)
**CENPM**	-0.81	Centromere protein M	Associated with the kinetochore complex (44)
**MELK**	-0.81	Maternal Embryonic Leucine Zipper Kinase (PEg3 kinase)	Regulates mitotic progression (45-47)
**NUSAP1**	-0.81	Nuclear and spindle associated protein 1	Organization of spindle microtubules (48)

Fig. S7. Cells of the 'posterior cluster' preferentially express genes involved in G2/M phases of the cell cycle. Correlation of gene expression with PC1 from scRNA-seq data. Of 37 genes with a correlation coefficient <0.80 for PC1, at least 31 are involved in G2/M phases of the cell cycle. Key cell-cycle-related roles of each gene outlined in column 4. Genes in red have been reported to be regulated by FOXM1, a specific transcriptional activator of G2/M phase related genes (which is itself represented among the genes in this group).

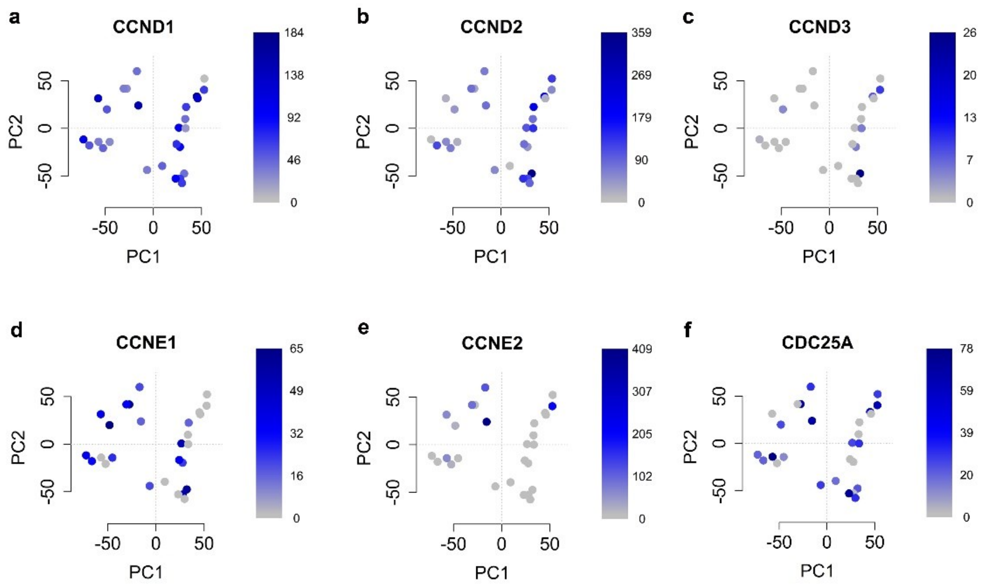

Fig. S8. Genes associated with G1/S phases of the cell cycle appear to be randomly distributed among node cells. The dataset comprises 27 cells collected from the HH8 node, originating from lateral epiblast grafts (as shown in Fig. 3d). Expression of a selection of cyclins **(a-e)** and CDC25A **(f),** all associated with the G1/S-phases of the cell cycle, are represented. FPKM levels reflected by intensity of blue. For the expression profile of G2/M cell cycle related genes associated with PC1 see 'Fig. 3f-g and Fig. S9j-m'.

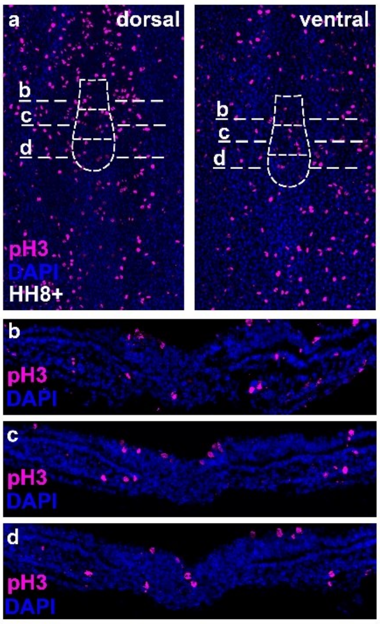

e Gene	FPKM			Fold change	
	A	M	P	P/M	P/A
PLK4	12	26	23	0.9	2.0
FOXM1	24	50	48	1.0	2.0
SMC2	188	376	370	1.0	2.0
NUSAP1	135	187	230	1.2	1.7
CDCA3	12	16	17	1.1	1.5
CENPO	23	30	34	1.2	1.5
HJURP	37	51	53	1.0	1.4
NCAPG	53	78	74	1.0	1.4
KIF15	54	71	74	1.0	1.4
PBK	214	220	290	1.3	1.4
RACGAP1	283	396	381	1.0	1.3
KIF11	92	111	123	1.1	1.3
TTK	82	96	109	1.1	1.3
CENPU	33	40	43	1.1	1.3
TOP2A	127	139	142	1.0	1.1
NUF2	217	217	240	1.1	1.1
CENPC	33	32	35	1.1	1.1
KIF2C	97	99	103	1.0	1.1
MELK	149	146	149	1.0	1.0
DEPDC1	22	21	22	1.0	1.0
SKA3	98	76	91	1.2	0.9
NCAPH2	11	8	10	1.3	0.9
HAUS1	186	160	157	1.0	0.8
CENPM	48	34	41	1.2	0.8
KNSTRN	187	161	157	1.0	0.8
CKS1B	630	490	523	1.1	0.8
BIRC5	555	387	458	1.2	0.8
CDK1	1142	864	880	1.0	0.8
MAD2L1BP	29	16	15	0.9	0.5
CENPL	12	6	5	0.8	0.4
CENPH	100	22	34	1.5	0.3

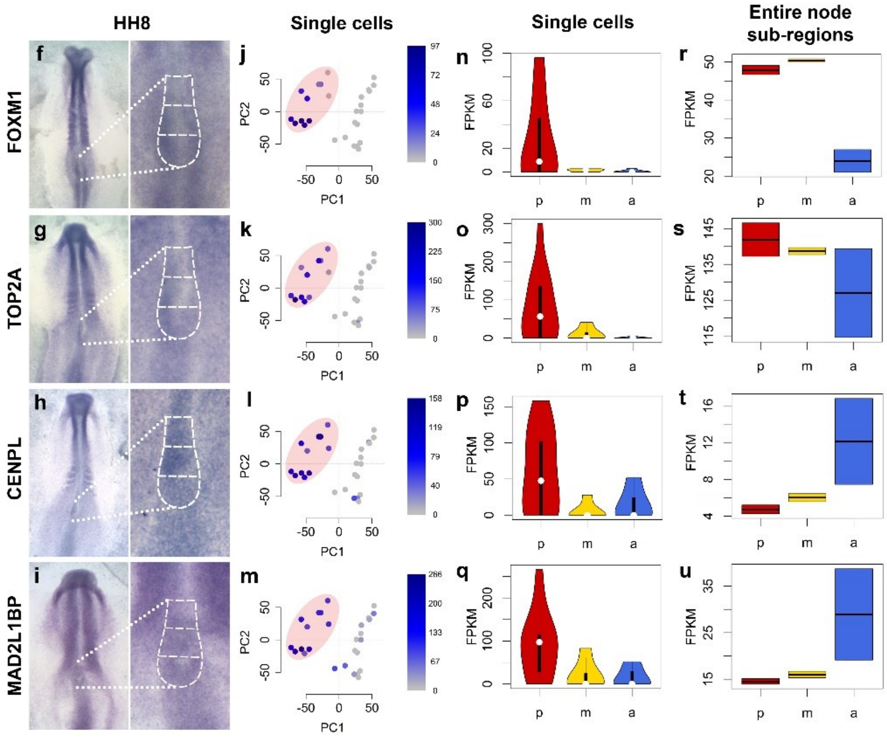

Fig. S9. Single resident cells express G2/M phase cell-cycle related genes in a distinctive manner; for some genes, this is different from the dominant pattern of expression in the same sub-region of the node. a-d, Distribution of dividing cells in the regressing node analysed using pH3 staining, showing no evident accumulation of actively dividing cells in the posterior node. Previously, G2/M phase cell cycle related genes were found to be enriched in single resident cells from the posterior node (Fig. S7). **e,** In the present table, expression of these G2/M cell cycle related genes is shown in entire node sub-regions obtained by bulk RNA-seq (A, anterior; M, middle; P, posterior). Red: fold

change >1; blue: fold change <1. Expression of genes highlighted in yellow also assessed spatially by *in situ* hybridization **(f-i)**, at the single cell level (scRNA-seq, dataset comprising 27 cells collected from the HH8 node, originating from lateral epiblast grafts, see Fig. 3d) **(j-q)** and then compared to expression in entire node sub-regions (bulk RNA-seq data) **(r-u)**. **f-i,** *in situ* hybridization shown in ventral view. **j-m,** Pink ovals show the cluster containing most cells derived from the posterior part of the node. FPKM levels reflected by intensity of blue. **n-q,** Violin plots show that the highest levels of G2/M phase cell cycle related genes are in single cells from the posterior (p, red) region of the node when compared to the middle (m) and anterior (a) regions. **r-u,** Boxplots show that the expression across entire node sub regions is also higher in the posterior node sub-regions for FOXM1 (r) and TOP2A (s), but is instead higher in the anterior node sub-regions for CENPL (t) and MAD2L1BP (u).

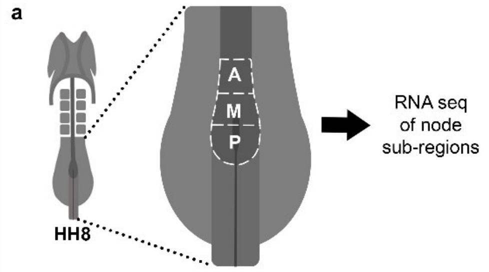

b

Gene	FPKM			Fold change	
	A	M	P	P/M	P/A
RSPO3	24.0	78.5	177.4	2.3	7.4
TBX6	22.7	34.4	116.8	3.4	5.1
CHST15	27.0	53.5	108.1	2.0	4.0
DLL1	38.2	63.9	150.5	2.4	3.9
AKAP12	17.1	34.7	65.1	1.9	3.8
MSGN1	40.4	34.6	101.7	2.9	2.5
DRAXIN	32.0	45.3	79.9	1.8	2.5
NKAIN4	34.3	31.4	74.3	2.4	2.2

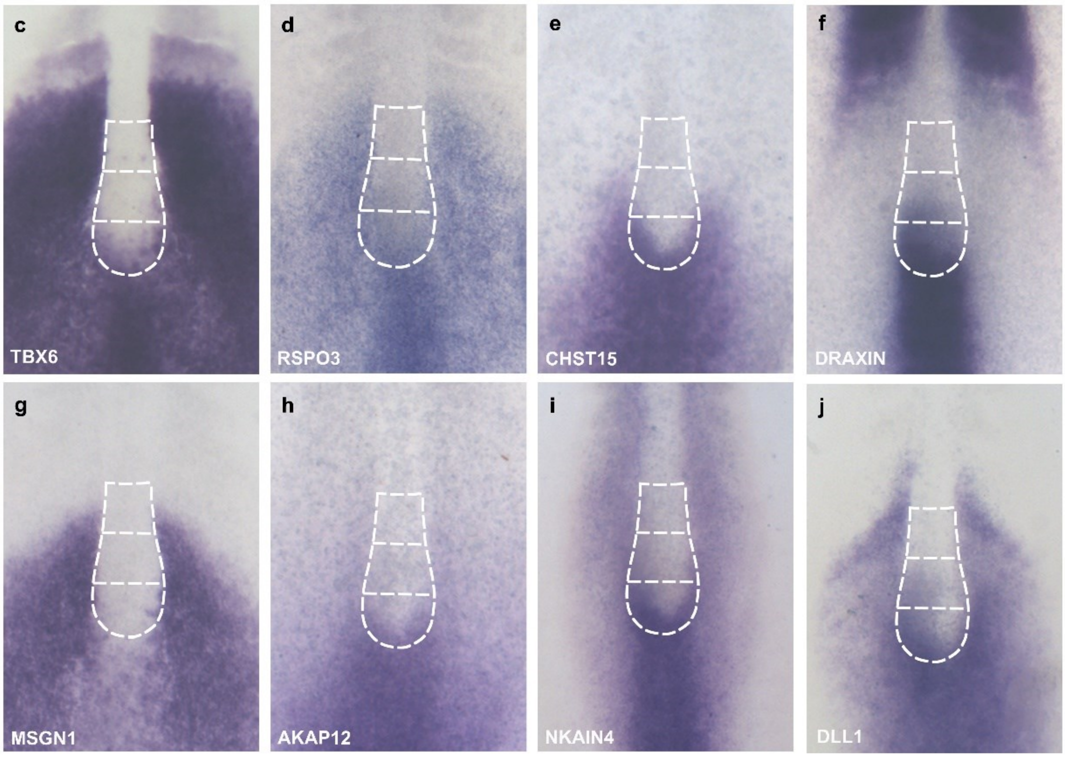

Fig. S10. Examples of genes with higher expression in posterior node, as revealed by RNA-seq of entire node sub-regions, verified by *in situ* hybridization. a, Node sub-regions sampled for bulk RNA-seq: A, anterior; M, middle; P, posterior. **b,** Genes with expression enriched in the posterior node (data from bulk RNA-seq). All selected genes have a fold-change of >1.7 in posterior versus anterior and middle regions, and have an FPKM value of >65 in in the posterior region. The intensity of red corresponds to degree of fold change. **c-j,** *In situ* hybridization of genes from (b). Anterior, middle and posterior regions of the node outlined. All embryos are between HH8- and HH8+, shown in ventral view. The pattern of expression within the posterior sub-region of the node can be 'salt-and-pepper' (c, g), 'diffuse' (d), 'horseshoe-like' (e, h, i) or 'asymmetrical' (f, j). Several of these genes are characteristic of different signaling pathways (Wnt, FGF and Notch) and/or have been implicated in stem cell niches in other systems (see main text).

a<br>Epiblast graft placed lateral to the HH3+/4 node	Tissue contribution of graft-derived cells after culture (no. and % of embryos)						
	Caudal node	CNH	Caudal node and/or CNH	Notochord	Floorplate	PSM/ medial somite	Lateral neural plate
**Epiblast lateral to the HH3+/4 node (n = 9)**	7/9 (78%)	7/9 (78%)	8/9 (89%)	8/9 (89%)	3/9 (33%)	9/9 (100%)	1/9 (11%)
**Epiblast far anterior to the HH3+/4 node (n = 34)**	29/34 (85%)	28/34 (82%)	30/34 (88%)	24/34 (71%)	15/34 (44%)	24/34 (71%)	9/34 (26%)
**Late (HH4+/5) epiblast (n = 11)**	4/11 (36%)	6/11 (55%)	6/11 (55%)	7/11 (64%)	5/11 (45%)	2/11 (18%)	3/11 (27%)

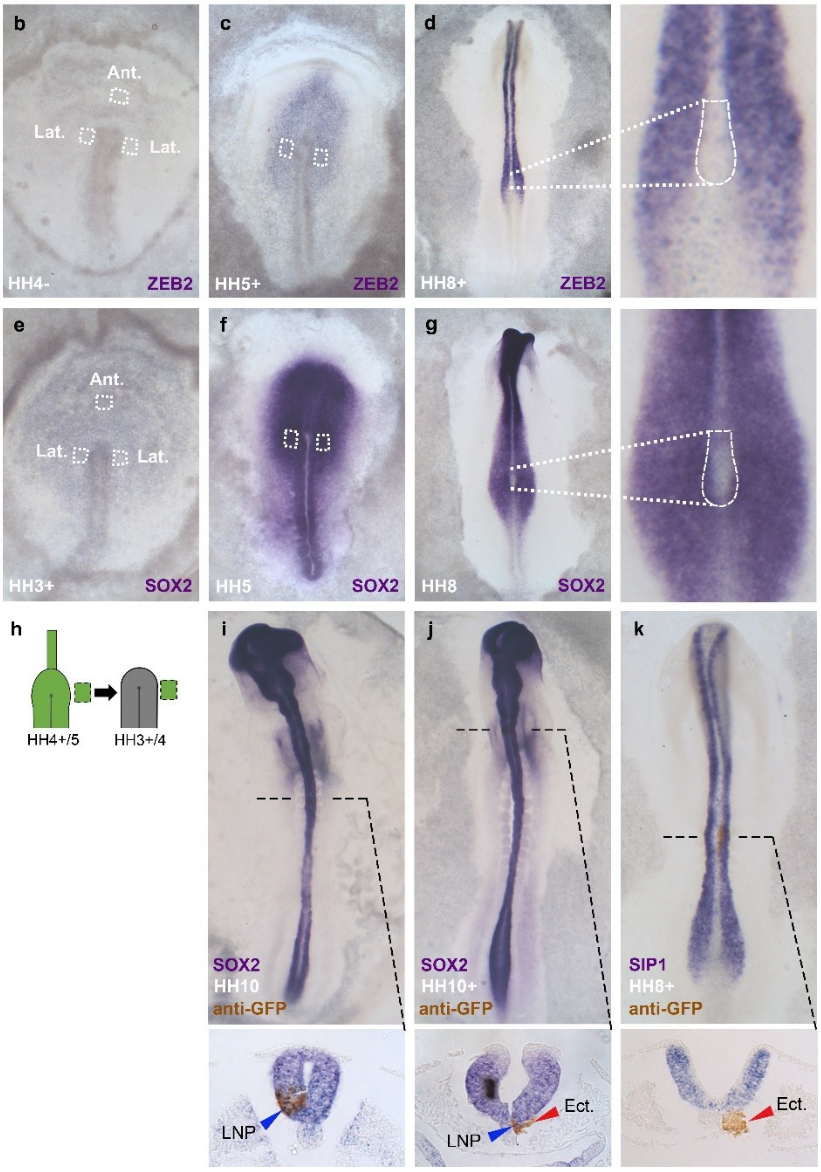

l<br>Stage of late-graft donor	Tissue contribution of graft-derived cells after culture (% of embryos)				
	Neural		Mesodermal		Node/CNH
	Medial floorplate	Lateral neural plate	Notochord	PSM/ medial somite	
**HH 4+ (n = 4)**	50	25	75	25	100
**HH 5- (n = 3)**	33	0	67	33	67
**HH 5 (n = 1)**	0	0	100	0	0
**HH 5+ (n = 3)**	67	67	33	0	0

Fig. S11. Late epiblast derived cells only express neural markers when in the neural plate. a, Table comparing the tissue contributions from epiblast grafts from late (HH4+/5) and early (HH3+/4) donor embryos. HH3+/4 donor epiblast grafts (green and blue) performed as shown in Fig. 1d and f. Late epiblast donor grafts (yellow) performed as in Fig. 4c. n-numbers indicate the number of embryos grafted. **b-g,** The neural plate markers ZEB2 and SOX2 are highly expressed in late (HH4+/5) donor epiblast (c, f) but absent or low in early (HH3+/4) donor epiblast (b, e). (Ant.: position from which anterior donor epiblast was taken; Lat.: region from which lateral donor epiblast was dissected). Expression in the regressing node is seen only in a few cells (d, g). All embryos shown in dorsal view. **h-k,** Late epiblast derived cells only express neural markers when in the neural plate. Late (HH4+/5) epiblast was grafted lateral to the HH3+/4 node (h). Resulting embryos after culture, processed for *in situ* hybridization and anti-GFP antibody staining (i-k). Whole mounts in dorsal view. The blue arrow indicates GFP-positive cells (brown) that overlap with the *in situ* signal (purple). The red arrow points to GFP-positive cells in ectopic locations not overlapping with the *in situ* signal. **l,** A transition away from mesodermal and towards neural fates in lateral epiblast occurs around stages HH5- to HH5. Epiblast grafts performed as in (h). n-numbers indicate the number of grafted embryos. The intensity of red reflects the proportion of embryos with graft-derived cells in the tissues indicated. CNH, chordoneural hinge; PSM, presomitic mesoderm; LNP, lateral neural plate; Ect., cells from the graft that failed to integrate into any host structure.

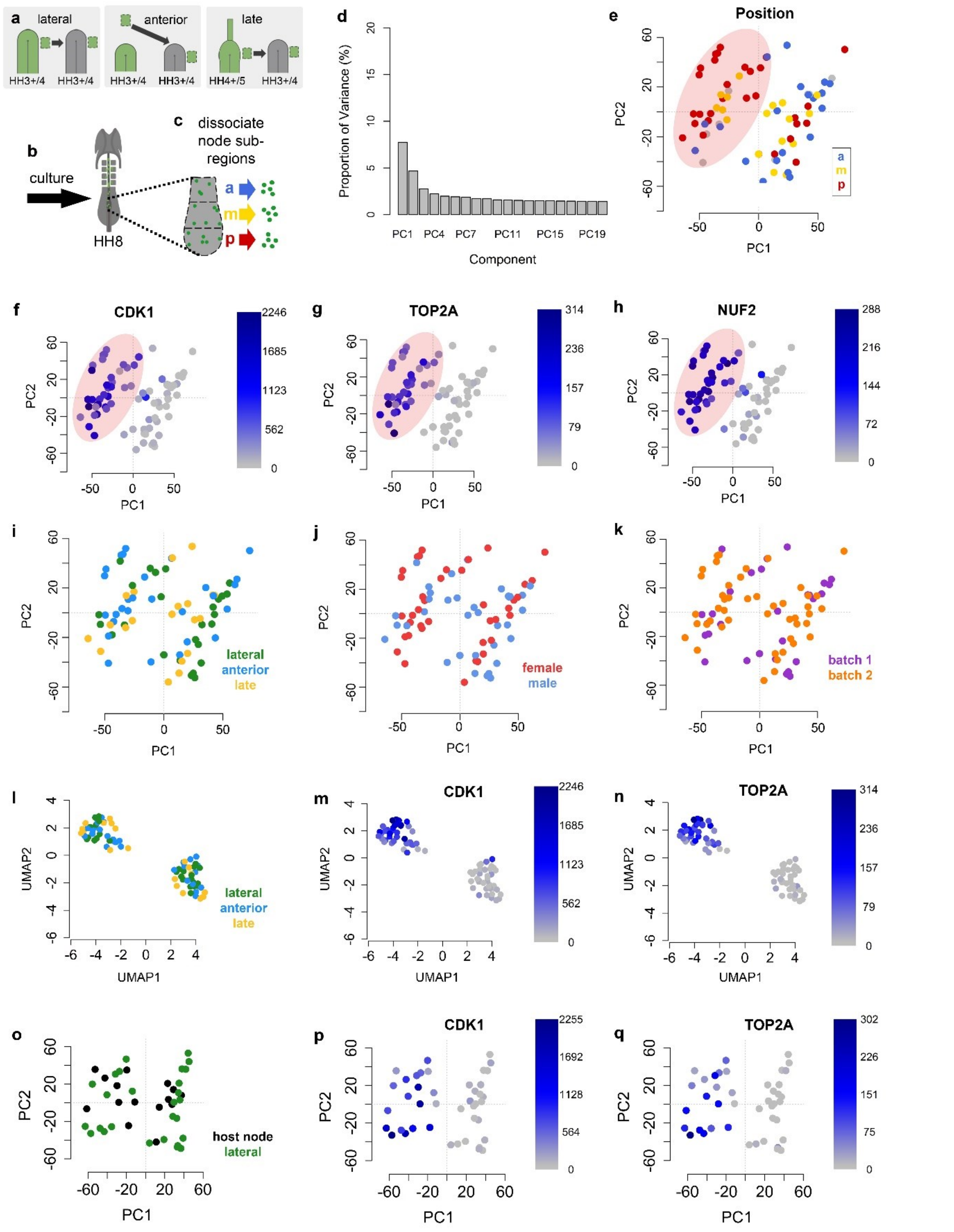

Fig. S12. Irrespective of a cell's origin, most variation among cells in the node is explained by expression of G2/M phase related cell-cycle genes. All single cells (dataset comprises 77 cells) were collected from the HH8 node but originate from three graft conditions. **a,** Epiblast just lateral to the node that normally enters the node ('lateral') and epiblast that was made to enter the node from 'anterior' or

‘late’(HH4+/5) epiblast. **b-c,** Grafted embryos were cultured to HH8 (b), the node dissociated and single GFP-positive cells isolated from anterior (a, blue), middle (m, yellow) and posterior (p, red) parts of the node and processed for scRNA-seq (c). **d,** Principal components (PCs) as a measure of variation in a dataset comprised of 77 cells collected from the HH8 node. The first 20 (out of 77) PCs are shown. The first three PCs provide the most informative measure for clustering. **e-k,** Cells plotted according to PC1-2. Pink ovals contain most cells from the posterior part of the node (‘posterior cluster’). The grey spots in (e) mark cells whose position could not be ascertained. Expression of G2/M phase cell-cycle related genes limited almost exclusively to cells in the ‘posterior cluster’ (f-h) (FPKM levels reflected by intensity of blue). Neither the cell’s origin (i.e. graft condition) (i)**,** nor its gender (j) nor the sequencing batch (k) correlate with PC1 or PC2. **l-m,** UMAP analysis groups cells into two clusters comparable to PC1-2 analysis (‘i’ versus ‘l’), with one cluster corresponding to cells expressing genes associated with the G2/M phases of the cell cycle (‘f-g’ versus ‘m-n’). **o-q,** PC analysis of lateral-derived node cells (n = 27) and non-grafted host node cells (n = 15) showing that some host cells are also enriched in G2/M phase cell-cycle related genes (dataset comprises 42 cells). Enrichment of G2/M phase cell-cycle related genes is therefore not an artefact of grafting.

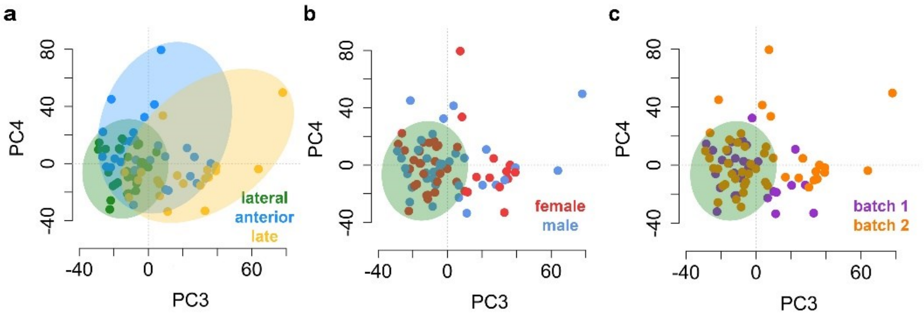

Fig. S13. Overlap with lateral-derived cells does not correlate with cell gender or sequencing batch. The dataset comprises 77 cells collected from the HH8 node, originating from three different graft conditions (as shown in Fig. 4f). PC3-4 groups cells into partially overlapping clusters according to graft condition **(a)**. Cells normally destined to enter the node (green) form a fairly tight cluster (green oval). Cell gender (male/female) (**b)** and sequencing batch **(c)** did not account for the lack of overlap of cells with the cluster of lateral-epiblast derived cells.

a Gene	PC3 correlation coefficient	Links to cell adhesion	Links to neural development
**CLDN1**	0.67	Component of tight junctions [99]	-
**PROCA1**	0.61	-	-
**PRTG**	0.61	Role in cell-cell adhesion during normal ingression of paraxial mesoderm [100]	Prevents early differentiation of neural progenitors [101]
-	0.60	-	-
**PHACTR1**	0.59	-	-
**LHFPL6**	0.58	-	-
**CSTA**	0.58	Protease inhibitor involved in cell adhesion [102]	-
**EDNRB**	0.58	-	Involved in normal colonization of the large intestine by enteric neural crest cells [103,104]
**RAB3IP**	0.58	-	-
**LIN28A**	0.58	-	Maintains neural cell progenitor pools by maintaining proliferation and preventing differentiation [105-107]
**HIC2**	0.56	-	-
**SLC39A3**	0.56	-	-
**NCAM1**	0.56	Cell-cell and cell-matrix adhesion that facilitates normal neural tube development [108,109]	
**PFKFB4**	0.56	-	
**PODXL**	0.55	Neuron-neuron adhesion and normal axonal growth [110]	

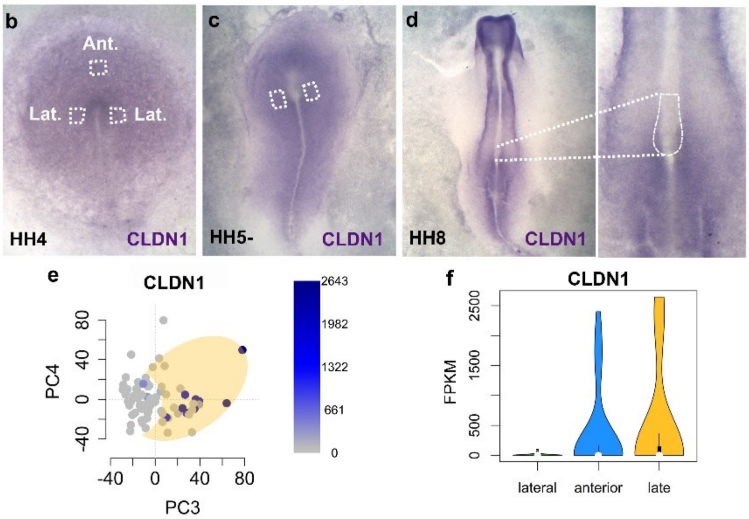

Fig. S14. Cells derived from late epiblast are characterized by a neural-plate-like gene expression signature. a, Correlation of gene expression with PC3 from scRNA-seq data. Of 15 genes with a correlation coefficient ≥0.55 for PC3, at least 7 are linked to cell adhesion and/or neural development. Expression of genes highlighted in yellow also assessed spatially (by *in situ* hybridization) and at the single cell level (this figure b-f and Fig. 4g-k). **b-c,** *In situ* hybridization for CLDN1 shows some expression in donor epiblast from early (HH3+/4) (b) and late (HH4+/5) (c) stage donor embryos. **d,** Expression is largely restricted to neural plate at the time of collection for scRNAseq. Ant.: position from which anterior donor epiblast was taken; Lat.: region from which lateral donor epiblast was dissected. All embryos shown in dorsal view. **e-f,** Expression of CLDN1 among single cells sequenced from the HH8 node (dataset comprises 77 cells) correlates with PC3 (e) and is restricted to cells originating from late epiblast (yellow) and anterior HH3+/4 epiblast (blue) (f). Yellow oval indicates late epiblast-derived cells not overlapping with the HH3+/4 lateral epiblast derived cells.

Single-cell re-grafts	All embryos after re-graft		Only embryos where positive anti-GFP antibody staining detected					
	Embryos with GFP-positive cells	Embryos with positive anti-GFP antibody staining	Embryos with positive anti-GFP antibody staining in more than one cell	Tissue contribution of GFP-positive cells after re-graft (no. and % of embryos)				
				Node	Midline	PSM/ somites	Head	GFP cells in node and axis
Single GFP-positive cell only (n = 39)	17/39 (44%)	**9/39 (23%)**	4/9 (44%)	2/9 (22%)	5/9 (56%)	1/9 (11%)	5/9 (56%)	2/9 (22%)
Single GFP-positive cell with GFP-negative cell/s attached (n = 36)	12/36 (33%)	**8/36 (22%)**	5/8 (63%)	3/8 (38%)	5/8 (63%)	1/8 (13%)	0/8 (0%)	1/8 (13%)
**TOTAL single GFP-positive cell re-grafts (n = 75)**	29/75 (39%)	**17/75 (23%)**	9/17 (53%)	5/17 (29%)	10/17 (59%)	2/17 (12%)	5/17 (29%)	3/17 (18%)

Fig. S15. Single GFP cells grafted alone versus single GFP-cells grafted with non-GFP neighbours. Table referring to single GFP-cell re-grafts performed as in Fig. 2. Here, tissue contributions from a single GFP-cell grafted alone are distinguished from those of a single GFP-cell grafted alongside one or more non-GFP neighbours (highlighted in red). A slight increase in contribution of GFP cells to node is seen when a GFP cell is grafted with non-GFP neighbours.

Movie S1 (separate file). Live tracking of cells in the node from HH5 to HH9. Time-lapse movie showing a mosaic of cells labelled with DsRed (pseudo-colour encoded as green). Selected cells originating from the anterior part of the node at HH5 are highlighted in blue and some originating from the posterior part of the node are highlighted in red. The outline of the node is highlighted by a white dashed line.

Dataset S1 (separate file). FPKM table for scRNA-seq data. Transcriptional profiling of single cells individually harvested from the HH8 node, originating from the three experimental conditions described in Fig. 4f, along with non-grafted node cells from the host. Details on the epiblast origin (developmental stages and position in the donor) of the cells and their positions within the host node at the time of collection are included in the raw data submitted to EBI Array Express (accession numbers E-MTAB-9116 and E-MTAB-11216).

Dataset S2 (separate file). FPKM table for bulk-RNA-seq data. Transcriptional profiling of 6 sub-regions of the HH8 node (see Fig. S10a). The raw data were submitted to EBI Array Express (accession number E-MTAB-9115). Abbreviations for the node regions at stage 8: S15_AL8: anterior-left; S16_AR8: anterior-right; S17_ML8: middle-left; S18_MR8: middle-right; S19_PL8: posterior-left; S20_PR8: posterior-right.

Supplementary Infornation References

1. L. Fang, A. Seki, G. Fang, SKAP associates with kinetochores and promotes the metaphase-to-anaphase transition. *Cell cycle* 8, 2819-2827 (2009).
2. N. Sugata *et al.*, Human CENP-H multimers colocalize with CENP-A and CENP-C at active centromere–kinetochore complexes. *Hum. Mol. Genet.* 9, 2919-2926 (2000).
3. N. Sugata, E. Munekata, K. Todokoro, Characterization of a novel kinetochore protein, CENP-H. *J. Biol. Chem.* 274, 27343-27346 (1999).
4. F. Li *et al.*, Control of apoptosis and mitotic spindle checkpoint by survivin. *Nature* 396, 580-584 (1998).
5. T. Hori, T. Haraguchi, Y. Hiraoka, H. Kimura, T. Fukagawa, Dynamic behavior of Nuf2-Hec1 complex that localizes to the centrosome and centromere and is essential for mitotic progression in vertebrate cells. *J. Cell Sci.* 116, 3347-3362 (2003).
6. J. Shaughnessy, Amplification and overexpression of CKS1B at chromosome band 1q21 is associated with reduced levels of p27 Kip1 and an aggressive clinical course in multiple myeloma. *Hematology* 10, 117-126 (2005).
7. N. G. Ayad *et al.*, Tome-1, a trigger of mitotic entry, is degraded during G1 via the APC. *Cell* 113, 101-113 (2003).
8. T. N. Gaitanos *et al.*, Stable kinetochore–microtubule interactions depend on the Ska complex and its new component Ska3/C13Orf3. *The EMBO journal* 28, 1442-1452 (2009).
9. J. R. Daum *et al.*, Ska3 is required for spindle checkpoint silencing and the maintenance of chromosome cohesion in mitosis. *Curr. Biol.* 19, 1467-1472 (2009).
10. S. Gaudet, D. Branton, R. A. Lue, Characterization of PDZ-binding kinase, a mitotic kinase. *Proceedings of the National Academy of Sciences* 97, 5167-5172 (2000).
11. S. Matsumoto *et al.*, Characterization of a MAPKK-like protein kinase TOPK. *Biochem. Biophys. Res. Commun.* 325, 997-1004 (2004).
12. S. Lawo *et al.*, HAUS, the 8-subunit human Augmin complex, regulates centrosome and spindle integrity. *Curr. Biol.* 19, 816-826 (2009).
13. M. B. Einarson, E. Cukierman, D. A. Compton, E. A. Golemis, Human enhancer of invasion-cluster, a coiled-coil protein required for passage through mitosis. *Mol. Cell. Biol.* 24, 3957-3971 (2004).
14. E. M. Dunleavy *et al.*, HJURP is a cell-cycle-dependent maintenance and deposition factor of CENP-A at centromeres. *Cell* 137, 485-497 (2009).
15. D. R. Foltz *et al.*, Centromere-specific assembly of CENP-a nucleosomes is mediated by HJURP. *Cell* 137, 472-484 (2009).
16. T. Hori, M. Okada, K. Maenaka, T. Fukagawa, CENP-O class proteins form a stable complex and are required for proper kinetochore function. *Mol. Biol. Cell* 19, 843-854 (2008).
17. A. V. Strunnikov, E. Hogan, D. Koshland, SMC2, a Saccharomyces cerevisiae gene essential for chromosome segregation and condensation, defines a subgroup within the SMC family. *Genes Dev.* 9, 587-599 (1995).
18. D. F. Hudson, P. Vagnarelli, R. Gassmann, W. C. Earnshaw, Condensin is required for nonhistone protein assembly and structural integrity of vertebrate mitotic chromosomes. *Dev. Cell* 5, 323-336 (2003).
19. T. Ono *et al.*, Differential contributions of condensin I and condensin II to mitotic chromosome architecture in vertebrate cells. *Cell* 115, 109-121 (2003).
20. D. Vanneste, M. Takagi, N. Imamoto, I. Vernos, The role of Hklp2 in the stabilization and maintenance of spindle bipolarity. *Curr. Biol.* 19, 1712-1717 (2009).
21. T. Habu, S. H. Kim, J. Weinstein, T. Matsumoto, Identification of a MAD2-binding protein, CMT2, and its role in mitosis. *The EMBO journal* 21, 6419-6428 (2002).
22. G. Xia *et al.*, Conformation-specific binding of p31comet antagonizes the function of Mad2 in the spindle checkpoint. *The EMBO journal* 23, 3133-3143 (2004).

23. K. Kimura, O. Cuvier, T. Hirano, Chromosome Condensation by a Human Condensin Complex inXenopus Egg Extracts. *J. Biol. Chem.* 276, 5417-5420 (2001).
24. G. Draetta *et al.*, Cdc2 protein kinase is complexed with both cyclin A and B: evidence for proteolytic inactivation of MPF. *Cell* 56, 829-838 (1989).
25. W. G. Dunphy, L. Brizuela, D. Beach, J. Newport, The Xenopus cdc2 protein is a component of MPF, a cytoplasmic regulator of mitosis. *Cell* 54, 423-431 (1988).
26. J. Gautier, C. Norbury, M. Lohka, P. Nurse, J. Maller, Purified maturation-promoting factor contains the product of a Xenopus homolog of the fission yeast cell cycle control gene cdc2+. *Cell* 54, 433-439 (1988).
27. C. Fode, C. Binkert, J. W. Dennis, Constitutive expression of murine Sak-a suppresses cell growth and induces multinucleation. *Mol. Cell. Biol.* 16, 4665-4672 (1996).
28. R. Habedanck, Y.-D. Stierhof, C. J. Wilkinson, E. A. Nigg, The Polo kinase Plk4 functions in centriole duplication. *Nat. Cell Biol.* 7, 1140-1146 (2005).
29. W. C. Earnshaw, N. Rothfield, Identification of a family of human centromere proteins using autoimmune sera from patients with scleroderma. *Chromosoma* 91, 313-321 (1985).
30. J. Tomkiel, C. A. Cooke, H. Saitoh, R. L. Bernat, W. C. Earnshaw, CENP-C is required for maintaining proper kinetochore size and for a timely transition to anaphase. *The Journal of cell biology* 125, 531-545 (1994).
31. W. Korver, J. Roose, H. Clevers, The winged-helix transcription factor Trident is expressed in cycling cells. *Nucleic Acids Res.* 25, 1715-1719 (1997).
32. J. Laoukili *et al.*, FoxM1 is required for execution of the mitotic programme and chromosome stability. *Nat. Cell Biol.* 7, 126 (2005).
33. G. B. Mills *et al.*, Expression of TTK, a novel human protein kinase, is associated with cell proliferation. *J. Biol. Chem.* 267, 16000-16006 (1992).
34. R. Schmandt, M. Hill, A. Amendola, G. B. Mills, D. Hogg, IL-2-induced expression of TTK, a serine, threonine, tyrosine kinase, correlates with cell cycle progression. *The Journal of Immunology* 152, 96-105 (1994).
35. A. Abrieu *et al.*, Mps1 is a kinetochore-associated kinase essential for the vertebrate mitotic checkpoint. *Cell* 106, 83-93 (2001).
36. D. Hogg *et al.*, Cell cycle dependent regulation of the protein kinase TTK. *Oncogene* 9, 89-96 (1994).
37. K. E. Sawin, K. LeGuellec, M. Philippe, T. J. Mitchison, Mitotic spindle organization by a plus-end-directed microtubule motor. *Nature* 359, 540-543 (1992).
38. L. Wordeman, T. J. Mitchison, Identification and partial characterization of mitotic centromere-associated kinesin, a kinesin-related protein that associates with centromeres during mitosis. *The Journal of cell biology* 128, 95-104 (1995).
39. M. Okada *et al.*, The CENP-H–I complex is required for the efficient incorporation of newly synthesized CENP-A into centromeres. *Nat. Cell Biol.* 8, 446-457 (2006).
40. Y. Mi *et al.*, DEPDC1 is a novel cell cycle related gene that regulates mitotic progression. *BMB reports* 48, 413 (2015).
41. K. Hirose, T. Kawashima, I. Iwamoto, T. Nosaka, T. Kitamura, MgcRacGAP is involved in cytokinesis through associating with mitotic spindle and midbody. *J. Biol. Chem.* 276, 5821-5828 (2001).
42. S. DiNardo, K. Voelkel, R. Sternglanz, DNA topoisomerase II mutant of Saccharomyces cerevisiae: topoisomerase II is required for segregation of daughter molecules at the termination of DNA replication. *Proceedings of the National Academy of Sciences* 81, 2616-2620 (1984).
43. T. Uemura *et al.*, DNA topoisomerase II is required for condensation and separation of mitotic chromosomes in S. pombe. *Cell* 50, 917-925 (1987).
44. D. R. Foltz *et al.*, The human CENP-A centromeric nucleosome-associated complex. *Nat. Cell Biol.* 8, 458-469 (2006).

45. J. Blot, I. Chartrain, C. Roghi, M. Philippe, J.-P. Tassan, Cell cycle regulation of pEg3, a new Xenopus protein kinase of the KIN1/PAR-1/MARK family. *Dev. Biol.* 241, 327-338 (2002).
46. N. Davezac, V. Baldin, J. Blot, B. Ducommun, J.-P. Tassan, Human pEg3 kinase associates with and phosphorylates CDC25B phosphatase: a potential role for pEg3 in cell cycle regulation. *Oncogene* 21, 7630-7641 (2002).
47. C. Badouel *et al.*, M-phase MELK activity is regulated by MPF and MAPK. *Cell Cycle* 5, 883-889 (2006).
48. T. Raemaekers *et al.*, NuSAP, a novel microtubule-associated protein involved in mitotic spindle organization. *The Journal of cell biology* 162, 1017-1029 (2003).
49. M. Furuse, K. Fujita, T. Hiiragi, K. Fujimoto, S. Tsukita, Claudin-1 and-2: novel integral membrane proteins localizing at tight junctions with no sequence similarity to occludin. *The Journal of cell biology* 141, 1539-1550 (1998).
50. K. Ito, H. Nakamura, Y. Watanabe, Protogenin mediates cell adhesion for ingression and re-epithelialization of paraxial mesodermal cells. *Dev. Biol.* 351, 13-24 (2011).
51. Y.-H. Wong *et al.*, Protogenin defines a transition stage during embryonic neurogenesis and prevents precocious neuronal differentiation. *J. Neurosci.* 30, 4428-4439 (2010).
52. D. C. Blaydon *et al.*, Mutations in CSTA, encoding Cystatin A, underlie exfoliative ichthyosis and reveal a role for this protease inhibitor in cell-cell adhesion. *The American Journal of Human Genetics* 89, 564-571 (2011).
53. R. P. Kapur, D. A. Sweetser, B. Doggett, J. R. Siebert, R. D. Palmiter, Intercellular signals downstream of endothelin receptor-B mediate colonization of the large intestine by enteric neuroblasts. *Development* 121, 3787-3795 (1995).
54. J. Amiel *et al.*, Heterozygous Endothelin Receptor B (EDNRB) Mutations in Isolated Hirschsprung Disease. *Hum. Mol. Genet.* 5, 355-357 (1996).
55. F. Cimadamore, A. Amador-Arjona, C. Chen, C.-T. Huang, A. V. Terskikh, SOX2–LIN28/let-7 pathway regulates proliferation and neurogenesis in neural precursors. *Proceedings of the National Academy of Sciences* 110, E3017-E3026 (2013).
56. M. Yang *et al.*, Lin28 promotes the proliferative capacity of neural progenitor cells in brain development. *Development* 142, 1616-1627 (2015).
57. D. A. Robinton *et al.*, The Lin28/let-7 Pathway Regulates the Mammalian Caudal Body Axis Elongation Program. *Dev. Cell* 48, 396-405. e393 (2019).
58. U. Rutishauser, A. Acheson, A. K. Hall, D. M. Mann, J. Sunshine, The neural cell adhesion molecule (NCAM) as a regulator of cell-cell interactions. *Science* 240, 53-57 (1988).
59. D. Newgreen, R. Kerr, J. Minichiello, N. Warren, Changes in cell adhesion and extracellular matrix molecules in spontaneous spinal neural tube defects in avian embryos. *Teratology* 55, 195-207 (1997).
60. N. Vitureira *et al.*, Podocalyxin is a novel polysialylated neural adhesion protein with multiple roles in neural development and synapse formation. *PLoS One* 5, e12003 (2010).
